# Polygenic subtype identified in ACCORD trial displays a favorable type 2 diabetes phenotype in the UKBiobank population

**DOI:** 10.1186/s40246-024-00639-z

**Published:** 2024-06-22

**Authors:** Courtney Hershberger, Arshiya Mariam, Kevin M. Pantalone, John B. Buse, Alison A. Motsinger-Reif, Daniel M. Rotroff

**Affiliations:** 1 Department of Quantitative Health Sciences, Lerner Research Institute, Cleveland Clinic, Cleveland, OH 44195 USA; 2 Center for Quantitative Metabolic Research, Cleveland Clinic, Cleveland, OH 44195 USA; 3 Endocrinology and Metabolism Institute, Cleveland Clinic, Cleveland, OH 44195 USA; 4 Division of Endocrinology, Department of Medicine, University of North Carolina at Chapel Hill, Chapel Hill, NC USA; 5 Biostatistics and Computational Biology Branch, National Institute of Environmental Health Sciences, National Institute of Health, Durham, NC USA

**Keywords:** Type 2 diabetes, A1c level, Single-nucleotide polymorphism (SNP), Genomics, Subtypes, Prescribing trends, Precision medicine, Polygenic score

## Abstract

**Introduction:**

We previously identified a genetic subtype (C4) of type 2 diabetes (T2D), benefitting from intensive glycemia treatment in the Action to Control Cardiovascular Risk in Diabetes (ACCORD) trial. Here, we characterized the population of patients that met the C4 criteria in the UKBiobank cohort.

**Research design and methods:**

Using our polygenic score (PS), we identified C4 individuals in the UKBiobank and tested C4 status with risk of developing T2D, cardiovascular disease (CVD) outcomes, and differences in T2D medications.

**Results:**

C4 individuals were less likely to develop T2D, were slightly older at T2D diagnosis, had lower HbA1c values, and were less likely to be prescribed T2D medications (*P* < .05). Genetic variants in *MAS1* and *IGF2R*, major components of the C4 PS, were associated with fewer overall T2D prescriptions.

**Conclusion:**

We have confirmed C4 individuals are a lower risk subpopulation of patients with T2D.

**Supplementary Information:**

The online version contains supplementary material available at 10.1186/s40246-024-00639-z.

## Introduction

The Action to Control Cardiovascular Risk in Diabetes Trial (ACCORD) sought to reduce cardiovascular disease (CVD) risk in individuals with type 2 diabetes (T2D) by lowering HbA1c levels to < 6% through intensive treatment. An observed increase in CVD events and mortality prematurely halted the intensive glycemia treatment arm, which greatly influenced T2D treatment [1]. We identified a subtype of patients (C4) in the intensive glycemia treatment arm that achieved and maintained the target HbA1c of < 6% and developed a polygenic risk score (PS SCT) to classify these individuals. The C4 group had significantly fewer CVD outcomes when treated intensively [2]. Notably, C4 individuals in the standard treatment arm of ACCORD did not display reduced CVD risk, indicating that the risk reduction in C4 occurred with intensive glycemia treatment [2]. The individuals in the C4 group had fewer years since their T2D diagnosis, slightly lower baseline HbA1c, and were less likely to use biguanides, sulfonylureas and thiazolidinediones, and insulin than non-C4 individuals [2]. In a genome-wide association study (GWAS), variants in *MAS1* and *IGF2R* nearly reached genome-wide statistical significance (*P* = 4.34 × 10^–7^) for association with C4 [2]. These single nucleotide polymorphisms (SNPs) were incorporated into a polygenic score (PS) to identify C4 individuals, in which they materially contribute to predicting C4 membership [2]. Notably, *MAS1* and *IGF2R* play an important role in cardiovascular function and glycemic response [3–6]. The UKBiobank has collected clinical and genetic data from ~ 500,000 participants [7, 8]. We sought to identify the C4 individuals in the UKBiobank and characterize this cohort as it relates to T2D onset and management.

## Research design and methods

### Polygenic score validation

The polygenic score (PS) for the identification of T2D patients responsive to intensive treatment was previously developed using ACCORD data [2]. Briefly, the stacking, clumping and thresholding algorithm (SCT) was used to derive scores from 178,674 SNPs [9]. Not all SNPs were included on both ACCORD and UKBiobank arrays (Concordance = 87.5%) (SNP *N* = 156,505). The exclusion of some SNPs from the arrays used on the ACCORD data caused the PS distribution to shift, thus requiring model threshold recalibration. We recalculated the threshold for C4 prediction in ACCORD training set using only the SNPs common in both ACCORD and UKBiobank. We recalibrated the model in ACCORD training set by identifying a new PS threshold (− 12.33) using only the common SNPs between both cohorts. The PS weights remained the same and only the threshold was changed (additional details in Supplemental Methods). This recalibrated model (UKB sctPS) was then applied to the ACCORD test set, and the UKBiobank cohort. The accuracy metrics for the recalibrated model are listed in Supplementary Table 1.

### Defining covariates and outcomes

A modified version of the Eastwood et al. algorithm was used to classify individuals with T2D in the UKBiobank [10] (Supplementary Fig. 1, Supplementary Methods). Other clinical variables were collected from surveys, nurse interviews, first assessments from the UKBiobank, ICD codes, and general practitioner prescription data (Supplementary Methods, Supplementary Tables 2–4). Seven SNPs within *MAS1* and 30 SNPs *IGF2R* were identified by UKBiobank whole exome sequencing with MAF ≥ 3%, a threshold which was found to appropriately control for genomic inflation [11]. These SNPs were tested for associations with the outcomes.

### Statistical tests

All statistical analyses was performed using R v.4.2.3 [12]. We tested for associations of C4 with T2D diagnosis using logistic regression, adjusting for BMI, age at first appointment, and sex. To test for associations of C4 with the age at T2D diagnosis, we used a Cox proportional hazard model, adjusting for BMI, and sex. Linear regression was used to test for associations of C4 with HbA1c mg/dL, adjusting for BMI, sex, and age at first appointment.

Time to CVD event analysis was performed using a Cox proportional hazard model and was used to test for associations of C4 with cardiac outcomes, while adjusting for BMI, age at time of event, and sex. We adjusted for multiple hypothesis testing within each cardiac outcome using a false discovery rate approach [13].

We used logistic regression to test for associations between C4 and any T2D prescriptions and ordinal logistic regression to test for associations with the number of unique T2D prescriptions. We further tested for associations between C4 within each class of T2D prescriptions using logistic regression followed by meta-analyzing the associations of each class of T2D prescriptions using a linear random-effects model. Each model was adjusted for BMI, sex, and age at last prescription, and *P-*values were adjusted for multiple hypothesis testing within each T2D prescription class [13].

We tested SNPs in *MAS1* and *IGF2R* for associations with C4, T2D diagnosis, age at diagnosis, HbA1c, cardiac events, T2D medications and drug classes, and the number of unique T2D medications using the logistic regression, linear random-effects meta-analysis, ordinal logistic regression, Cox proportional hazard model, and linear regression tests described above, with the same adjustments for multiple hypothesis testing. We adjusted for the previously described covariates, and for population substructure by incorporating the first 10 principal components as covariates.

## Results

### C4 is associated with a favorable T2D phenotype

**Fig. 1:**
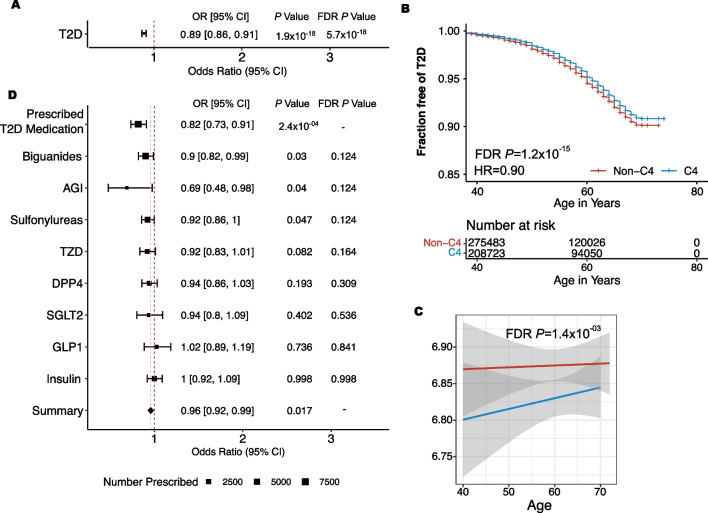
Associations of C4 with T2D Outcomes. **A** C4 compared to non-C4 using a generalized linear model to test associations with type 2 diabetes (FDR *P* = 5.7 × 10^–18^). **B** Time to T2D diagnosis was assessed using a Cox proportional hazard model (FDR *P* = 1.2 × 10^–15^). **C** HbA1c was compared between C4 and non-C4 using linear regression (FDR *P* = 1.4 × 10^–03^). **D** C4 individuals are less likely to be prescribed T2D medications (*P* = 2.3 × 10^–4^, OR = 0.82)

In the UKBiobank, 43.06% of the entire cohort (C4 = 210,152/488,001), and 40.06% of individuals with T2D (C4 = 9927/24,580), were classified as C4 using the UKB sctPS (UKBiobank recalibrated sctPS). In the UKBiobank, C4 individuals were less likely to be diagnosed with T2D (*P* = 1.89 × 10^–18^, OR = 0.89, C4 with T2D = 9927(4.7%), non-C4 with T2D = 14,653(5.3%) (Fig. 1A). Among the 24,580 individuals with T2D, the C4 subgroup was diagnosed at a slightly later age than the non-C4 group (FDR *P* = 8.44 × 10^–16^, HR = 0.90) (Fig. 1B). The C4 subgroup also had a lower HbA1c (mg/dL) (FDR *P* = 1.44 × 10^–03^, Beta = − 0.05) at the time of their first appointment (Fig. 1C).

The UKBiobank is comprised of 94% white individuals, whereas the ACCORD trial was 62% white. The UKB sctPS was calibrated on the entire ACCORD cohort. While 40.06% of individuals with T2D in the UKBiobank were predicted to be C4, in the full ACCORD cohort, only 31.71% of individuals were predicted to be C4. However, when the ACCORD cohort was subset by white race and the UKB sctPS was applied, 42.48% of individuals were predicted to be C4.

### ACCORD and UKBiobank CVD outcomes using recalibrated SCT PS

When the UKB sctPS was applied to the original ACCORD cohort, among all C4 individuals, the intensively treated test and training sets showed decreased risk cardiac outcomes when compared to those receiving standard care (Training *P* = 2e10^−5^, Test *P* = 0.002) (Supplementary Fig. 2) this decreased risk was also observed in the white C4 cohort (Training *P* = 3e10^−6^, Test *P* = 0.004) (Supplementary Fig. 3). Among all the individuals in the standard glycemia arm in ACCORD, there were no statistically significant differences in adverse events between predicted C4 and non-C4 groups using the original PS [2]. However, using the UKB sctPS, the predicted C4 group showed marginal increases in coronary heart disease (*P* = 0.03), macrovascular events (*P* = 0.002), and fatal myocardial infarction (MI) (*P* = 0.042) on standard glycemia treatment (Supplementary Fig. 4).

In the UKBiobank, of the 23,067 individuals with T2D and ICD codes available in the UKBiobank, 9332 were predicted to be C4. Within this subset, no associations were observed between the C4 subtype and time to CVD event (Supplementary Table 5).

### C4 is associated with fewer prescriptions for T2D medications

Medication data was available for 10,990 individuals diagnosed with T2D in the UKBiobank (C4 = 4524). Based on these records, fewer C4 individuals were prescribed T2D medications when compared to non-C4 individuals (*P* = 2.3 × 10^–4^, OR = 0.82) (Fig. 1D). Of the individuals in the C4 subgroup that were prescribed T2D medications, there was no significant difference in the number of unique medications prescribed, compared to the individuals in the non-C4 group (Supplementary Table 6). There were no significant differences between individual classes of T2D medication, including insulins, biguanides, sulfonylureas, AGIs, DPP4 inhibitors, GLP1 inhibitors, SGLT2 inhibitors or TZDs after multiple hypothesis correction (FDR > 0.05). However, a meta-analysis of these associations showed a significant decrease across the prescription classes (*P* = 0.017) (Fig. 1D).

### MAS1 and IGF2R SNPs are associated with C4

**Fig. 2:**
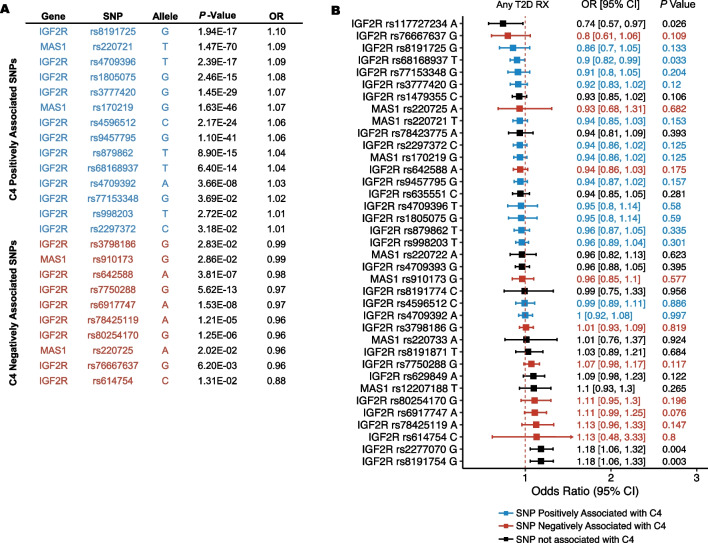
Associations of *MAS1* and *IGF2R* SNPs with C4 and T2D Prescriptions. **A** *IGF2R* and *MAS1* SNPs significantly associated with C4, colored by the odds ratio. **B** Odds ratio describing the likelihood of being prescribed any T2D medications for each SNP colored by the association with the C4 subtype

Combined Annotation Dependent Deletion (CADD) scores for the 37 SNPs assessed in this study ranged from 0.001 to 22.5 (mean = 3.84 and median = 2.23) (Supplementary Table 7). Four SNPs in *MAS1* and 20 SNPs in *IGF2R* were significantly associated with C4 (*P* < 0.05) (Fig. 2A, Supplementary Table 7). *IGF2R and MAS1* SNPs were not significantly associated with T2D occurrence, time to T2D diagnosis, nor HbA1c (FDR < 0.05) (Supplementary Tables 8–9).

### IGF2R SNPs are associated with an increased risk for CVD outcomes

*IGF2R* SNPs rs635551 C and rs8191871 T were associated with an increased risk of CVD (FDR *P* = 0.02, OR 1.068) and an increased risk of MI (FDR *P* = 0.04, OR = 1.15), respectively (Supplementary Table 10).

### MAS1 and IGF2R SNPs are associated with T2D prescription patterns

Four *IGF2R* SNPs associated with C4 were significantly associated altered likelihood of being prescribed T2D medications (*P* < 0.05) (Fig. 2B). Furthermore, of the individuals who were prescribed T2D medications, two *MAS1* SNPs and six *IGF2R* SNPs were also significantly associated with differences in the number of unique T2D medication prescriptions. (Supplementary Table 11).

Those with T allele for *MAS1* rs220721 were less likely to be prescribed sulfonylureas (FDR *P* = 0.04, OR = 0.91) and those with the G allele of *IGF2R* rs8191754 were more likely to be prescribed biguanides (FDR *P* = 0.04, OR = 1.15) (Supplementary Table 12).

## Discussion

We characterized the PS-predicted C4 subtype in the UKBiobank. While the ACCORD cohort was comprised of individuals who were high risk for CVD outcomes, the UKBiobank cohort is healthier than the general population [14].

Importantly, among C4 individuals in ACCORD, only those treated intensively for glycemia (target HbA1c < 6%) exhibited a decreased risk of CVD events, demonstrating a beneficial interaction between this subtype and intensive treatment. Individuals in the ACCORD cohort on standard glycemia treatment even displayed marginal increases in coronary heart disease (*P* = 0.03), and fatal MI (*P* = 0.042), after classification using the UKB sctPS. In the UKBiobank cohort, we also observed increased risk of CVD (FDR *P* = 0.02) and an increased risk of MI (FDR *P* = 0.04) for those with *IGF2R* SNPs rs635551 C and rs8191871 T alleles, respectively.

It is unlikely that the individuals in the UKBiobank were treated intensively for glycemia, and the mean HbA1c value at the time of the first appointment was 6.9% mg/dL in this cohort. Therefore, it is not surprising that modified risk of cardiac events was not observed in C4 individuals in the UKBiobank. These results support that belonging to the C4 subtype alone does not decrease risk for CVD outcomes, highlighting the need for investigation into the interaction of intensive treatment in the C4 subtype in order to reduce cardiac events.

*MAS1* and *IGF2R* SNPs positively associated with C4 were also most often associated with favorable T2D prescription patterns. Of the two *MAS1* and 12 *IGF2R* SNPs that were significantly associated with C4 membership, *IGF2R* rs68168937 T was associated with a decreased likelihood of being prescribed T2D medication, *IGF2R* rs3777420 G, *MAS1* rs220721 T, *MAS1* rs170219 G were associated with being prescribed fewer unique T2D medications, and *MAS1* rs220721 T was associated with decreased likelihood of being prescribed sulfonylureas (Fig. 2B). Only *IGF2R* rs998203 was associated with being prescribed a greater number of unique T2D medications.

CADD scores estimate the deleteriousness of SNPs. Four *IGF2R* SNPs had CADD scores greater than 10, indicating that they were in the top 10% of deleterious SNPs. Two of these were associated with T2D prescribing patterns: missense variant *IGF2R* rs8191754 G (CADD = 22.5) and SNP *IGF2R* rs68168937 T, which is located in an intronic regulatory region. Five *IGF2R* SNPs and one *MAS1* SNP had CAD scores greater than five. Of these, three were significantly associated with T2D prescription patterns (*IGF2R* rs117727234 A, rs3777420 G, rs78423775 A) and one was associated with increased risk of MI (*IGF2R* rs8191871 T).

*IGF2R *and *MAS1* function at the intersection of cardiac and metabolic health and may play a role in response to T2D treatment. *IGF2R* encodes a receptor for insulin-like growth factor type 2 (IGF2) and mannose 6-phosphate. Circulating *IGF2R* is associated with insulin resistance [6] and the rs416572 C allele has been previously shown to be associated with T2D diagnosis [6]. In addition to playing a role in glucose homeostasis, IGF2R protein expression was upregulated under high glucose conditions in hearts of a diabetic rat model, resulting in activation of cardiac hypotrophy and apoptosis proteins, resulting in cardiomyocyte apoptosis [8]. As part of the Renin-Angiotensin System, *MAS1* is the receptor for Ang-(1-7), a cleaved product of angiotensin I. Ang-(1-7) has been shown to promote vasodilation through AKT and the release of nitric oxide [3, 15]. In rodent models, Ang-(1-7) has been shown to improve cardiac function after cardiac events [16–18]. *Mas1* is also implicated in metabolic disorders, and a knockout mouse model displays impaired glucose tolerance and increased body weight [4, 5, 19].

Overall, we have characterized a previously identified genetic subtype of T2D from the ACCORD clinical trial. In the broader real-world data from the UKBiobank population, this subtype was 11% less likely to be diagnosed with T2D and were diagnosed at a slightly later age (HR = 0.90). Among those with T2D, individuals in this genetic subtype had lower HbA1c, and were less likely to be prescribed T2D medication, indicating they may require less treatment to maintain glucose homeostasis, and *IGF2R* and *MAS1* variants may contribute to this phenotype. These findings confirm opportunities for precision medicine to define treatment of T2D.

### Supplementary Information

Supplementary Material 1.

## Data Availability

No datasets were generated or analysed during the current study.
