# Supplementary material for: Polygenic subtype identified in ACCORD trial displays a favorable type 2 diabetes phenotype in the UKBiobank population
Source: Hum Genomics. 2024 Jun 22;18:70. doi: 10.1186/s40246-024-00639-z (PMC11193210; doi:10.1186/s40246-024-00639-z)
Supplement: Supplementary file 1 — Supplementary Material 1. [file 40246_2024_639_MOESM1_ESM.docx]

**Supplementary Material**

**Supplementary Methods**

*Algorithm for identifying individuals with T2D (Supplementary Figure 1)*

Tier 1

1.1 Of the 488,001 individuals in the cohort, 27,120 individuals self-reported a diabetes diagnosis (type 1 or 2) in the nurse interview, or self-reported insulin or diabetes medications in the nurse interview or on the touch screen or, reported both female sex and gestational diabetes. Individuals who did not meet this criteria were classified as “T2D unlikely.”

1.2 Individuals who likely had gestational diabetes but not type 1 or type 2 diabetes were identified if they only reported gestational diabetes, did not self-report type 1 or type 2 diabetes, were over 50 (NI/TS), and had no report of insulin or non-metformin diabetes medications (NI). These individuals were classified as “T2D unlikely.”

1.3 Individuals who were prescribed a non-metformin oral diabetes medication were categorized as “possible T2D.”

1.4 Individuals who were not taking a non-metformin oral diabetes medication (NI) may have Type 1 diabetes. Therefore, these individuals were classified as “possible T2D” if they reported an age of diagnosis greater than 30 years old (TS/NI).

1.5 Individuals diagnosed under 30, who did not self-report current insulin use (TS), nor T1D diagnosis (NI) were also categorized as “possible T2D.”

Tier 2 Those categorized as “possible T2D” were filtered through a second algorithm.

2.1 Individuals who were prescribed metformin but not any other diabetes medications or insulin (NI) were removed unless (2.2) they self reported any type of diabetes (NI).

2.3 Of the individuals remaining, those who were taking a non-metformin oral diabetes medication (NI) were categorized as T2D.

2.4 Individuals who had self-reported diabetes diagnosis, but were not prescribed diabetes oral medications, and were taking insulin, were removed if they had self-reported type 1 diabetes. The remaining individuals were classified as T2D.

*Defining covariates and outcomes*

At the first visit, body mass index and glycated hemoglobin blood biochemistry were measured. Birth month, birth year, sex, and ethnicity were also recorded. Variables from the nurse interview included self-reported non-cancer illness with date of diagnosis, and self-reported diabetes medications. Variables incorporated from the touch screen interview included diabetes self-report, age at diabetes diagnosis, self-reported gestational diabetes, and self-reported insulin medication. Additionally, Readcodes for T1D (E10) and T2D (E11) were used in the classification of individuals.

*Estimation of Age covariates*

The estimated birthday was defined as the first of the month in which the individual was born. From these variables, we calculated the age at appointment 1 as the time difference between the estimated birthday and the date of the first appointment. We calculated the date of diagnosis as the time difference between the estimated birthday and the age at T2D diagnosis. Time with T2D was calculated as the time difference between the age at appointment 1 and the age at diagnosis. The age at last ICD record was the time difference between the latest ICD10 code for the whole cohort (02/02/2021) and each individual’s estimated birthday. The age at last prescription record was calculated as the time difference between the last prescription record for the whole cohort (3/31/2016) and each individual’s birthday.

*Defining cardiac outcomes*

The international classification of disease ICD-9 and ICD-10 codes were used to define acute myocardial infarction, acute stroke, cardiovascular disease, cerebrovascular disease and heart failure as previously described^1^ (Supplementary Table 2).

*Medication coding*

The Ukbiobank provided general practitioner prescriptions for 10,990 individuals in the T2D cohort. Each prescription had a combination of medication name and/or BNF code and/or Read 2 code. The Ukbiobank provides a database that translates Read2 codes to BNF codes (all_lkps_maps_v3).

There were 34,856 unique prescriptions for the 10,990 individuals in our cohort. 27,920 unique prescriptions had either BNF codes or Read2 codes. Read2 codes were translated to BNF codes. Prescriptions with BNF codes were subset for Chapter 06, Section 01, Paragraph 01 or 02. We classified 1,989 prescriptions as medications to treat diabetes and 25,923 prescriptions as medications that were not used to treat diabetes.

There were 6,936 remaining prescriptions had no BNF codes or Read2 codes and needed to be sorted by medication name or term description. Using the BNF dictionary and UKBiobank prescription names and descriptions of the medications that were already identified as used to treat T2D, we curated a list of 682 T2D prescription names, terms and keywords. Of these, 104 T2D keywords were matched to either the prescription name or the medication description (Supplementary Table 3).

Using BNF codes, Read 2 codes and this list of terms for pattern matching, we classified 2,219 medications that were prescribed to treat T2D.

Prescription information was also provided in the nurse interview (field = 20003.0). The ATC database 2019 was used to identify diabetes medications (code A10). Any medications recorded in the nurse interview that matched the terms in the ATC database were classified accordingly. These medications were further divided into metformin, AGI, TZD, sulfonylureas, biguanides and DPP4.

Insulin usage was collected in the touch screen questionnaire (field 6153 and 6177).

*Polygenic score validation*

The polygenic score (PS) for the identification of T2D patients responsive to intensive treatment was previously developed using ACCORD data.^2^ Briefly, the stacking, clumping and thresholding algorithm (SCT) was used to derive scores from 178,674 SNPs in ACCORD intensive treatment arm.^7^ SCT leverages GWAS summary statistics to calculate PS and a PS threshold was identified to classify individuals to C4. However, UKBiobank array only captured 156,505/178,674 (87.5%) of ACCORD SNPs and these missing SNPs resulted in shifting of PS such that the original PS threshold (-0.61) for identifying C4 was no longer applicable. We recalibrated the model in ACCORD training set by identifying a new PS threshold (-12.33) using only the common SNPs between both cohorts. The SNPs and their PS weights remained the same and only the threshold was changed. The accuracy metrics for the recalibrated model are listed in Supplementary Table 4. This recalibrated model was then applied to the ACCORD test set, and the UKBiobank cohort.

Supplementary Results

*ACCORD: C4, identified by the recalibrated PS, showed reduced cardiac events when subjects were treated with intensive glycemic control (A1C target<6%)in the ACCORD cohort.*

The recalibrated PS detected individuals in the C4 subtype with a sensitivity of 0.806, a specificity 0.918, and an AUC of 0.86 in the ACCORD training set (Supplementary Table 4). In the ACCORD test set, the recalibrated PS detected C4 with an AUC of 0.804 and a sensitivity and specificity of 0.741 and 0.867, respectively (Supplementary Table 4).

Using the recalibrated PS, 363/1177 (30.84%) of the intensive treatment ACCORD test set were predicted to be C4. In the standard arm of the trial, 1277/4027 individuals (31.71%) were predicted to belong to the C4 subtype. Among only white individuals, 325/765 (42.48%) in the intensive treatment arm and 1172/2598 (45.11%) of individuals in the standard treatment arm were predicted to be C4.

Among all C4 individuals, the intensively treated test and training sets showed decreased risk cardiac outcomes when compared to those receiving standard care (Training *P*=2e10^-5^, Test *P*=.002). The intensively treated C4 test set specifically showed decreased risk of coronary heart disease (*P*=.046), myocardial infarction (*P*=.043), and MACE (*P*=.005) when compared to the standard treatment C4 group (Supplementary Figure 2). Similar to the full cohort, MACE (P=.009) and coronary heart disease (P=.047) showed decreased risk in the intensively treated C4 group when compared to C4 individuals in the standard treatment arm. Among individuals in the standard glycemia arm in ACCORD, there were no statistically significant differences in adverse events between predicted C4 and non-C4 groups using the original PS.^2^ However, using the recalibrated PS, the predicted C4 group showed marginal increases in coronary heart disease (*P*=.03), macrovascular events (*P*=.002), and fatal myocardial infarction (*P*=.042) on standard glycemia treatment (Supplementary Figure 4).

**Supplementary Figures**

**Supplementary Figure 1:** Filtering steps describing the classification of individuals with T2D in the UKBiobank.





**Supplementary Figure 2:** Cardiovascular risk in the UKB sctPS C4 intensive treatment group compared to UKB sctPS C4 standard treatment group in the training and test ACCORD trial cohorts.


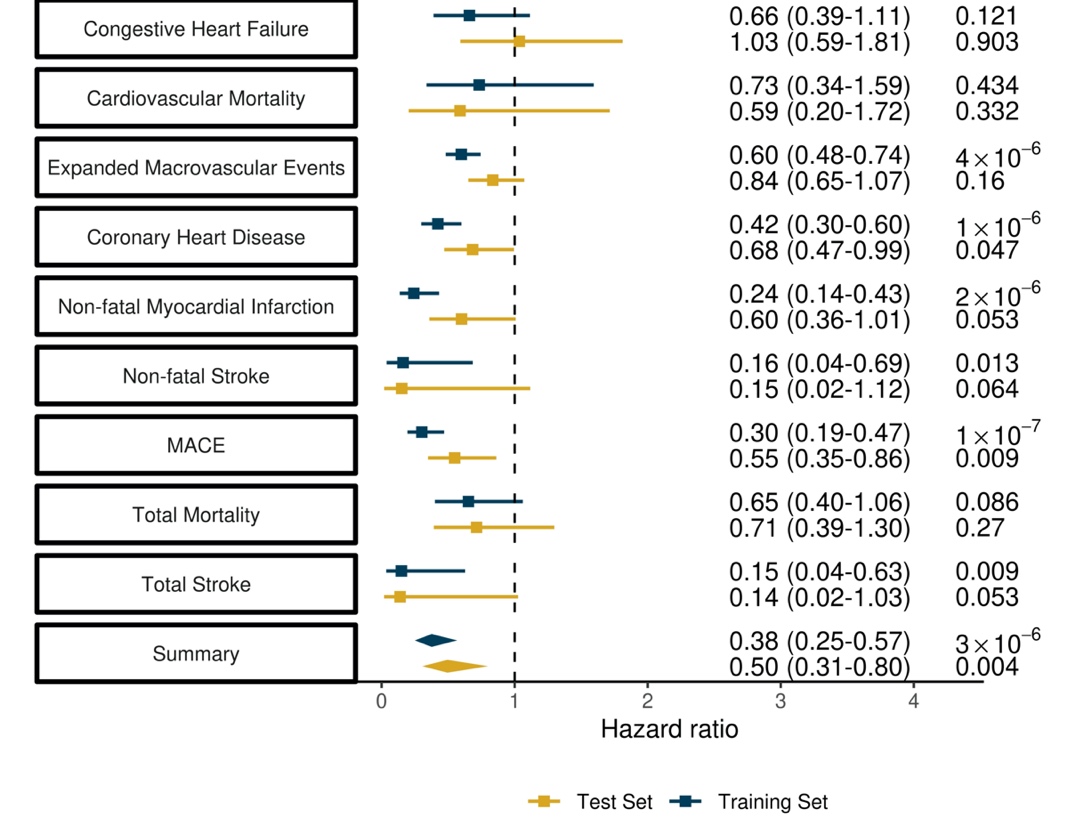


**Supplementary Figure 3:** Cardiovascular risk in the UKB sctPS white-C4 intensive treatment group compared to UKB sctPS white-C4 standard treatment group in the training and test ACCORD trial cohorts.

*

*

**Supplementary Figure 4:** Cardiovascular risk in C4 compared to non-C4 among individuals in the standard treatment group of the ACCORD trial cohort. Red indicates that the UKB sctPS was used to classify C4 and green indicates that the previously published ACCORD sctPS was used to classify C4.

**Supplementary Tables**

**Supplementary Table 1:** The accuracy metrics of the recalibrated sctPS using a threshold of -12.333 for classifying C4 individuals across the entire cohort (ACCORD), and the test and train subsets, as described in our previous publication^1^.

| **Threshold** | **AUC** | **Sensitivity** | **Specificity** | **Balanced Accuracy** | **ACCORD Cohort** |
| --- | --- | --- | --- | --- | --- |
| -12.333 | 0.843 | 0.784 | 0.901 | 0.843 | ACCORD |
| -12.333 | 0.805 | 0.741 | 0.867 | 0.804 | Test |
| -12.333 | 0.862 | 0.806 | 0.918 | 0.862 | Train |

**Supplementary Table 2:** UKBiobank Data-Fields and calculations for outcomes and covariates.

| **Variable** | **Data-Field** | **Description** |
| --- | --- | --- |
| BMI | 21001 | Body Mass Index at first visit |
| HbA1c | 30750 | Glycated haemoglobin Blood biochemistry at first visit |
| Date appointment 1 | 53 | Date of attending assessment centre |
| Estimated Birthday | Month=52 Year=34 | month of birth, year of birth |
| Sex | 31 | Sex |
| Ethnicity | 21000 | Ethnic Background |
| Medical Conditions (NI) | 20002 | Non Cancer Illness, self-reported medical conditions (NI date, unless missing, then TS date) |
| Age at diagnosis (NI) | 20009 | Interpolated age of participant when non-cancer illness first diagnosed |
| PCs | 22009.0.1-22009.0.10 | First 10 Principal Components |
| Diabetes Self-Report (TS) | 2443 | Diabetes diagnosed by doctor |
| Age Diabetes Diagnosis (TS) | 2976 | Age diabetes diagnosed medical conditions |
| Gestational Diabetes Self-Report (TS) | 4041 | Gestational diabetes only |
| Readcode T1D | 130707 | Source of report of E10 (insulin-dependent diabetes mellitus) |
| Readcode T2D | 130709 | Source of report of E11 (non-insulin-dependent diabetes mellitus) |
| Self-Report Insulin (TS) | 6153, 6177 | Medication for cholesterol, blood pressure, diabetes or take exogenous hormones |
| Self-Report Diabetes Medications (NI) | 20003 | Treatment/medication code: ATC A10, "metformin\|fortamet\|glucophage\|glumetza\|riomet” |

**Supplementary Table 3:** ICD-9 and ICD-10 Codes for Cardiac Outcomes

| **type** | **ICD-9 Codes** | **ICD-10 Codes** |
| --- | --- | --- |
| Acute MI | 4109 | I212, I211, I213, I214, I219, I210, I248, I249, I241, I240 |
| Acute Stroke | 4330, 4331, 4332, 4333, 4338, 4339, 4340, 4341, 4349 | I619, I630, I635, I632, I633, I631, I634, I652, I651, I658, I650, I659, I661, I669, I662, I660 |
| CVD | 2891, 2892, 2893, 4150, 4160, 4161, 4168, 4169, 4170, 4171, 4178, 4179, 4200, 4209, 4210, 4211, 4219, 4220, 4229, 4230, 4231, 4232, 4238, 4239, 4240, 4241, 4242, 4243, 4249, 4250, 4251, 4252, 4253, 4254, 4255, 4258, 4259, 4260, 4261, 4262, 4263, 4264, 4265, 4266, 4267, 4268, 4269, 4270, 4271, 4272, 4273, 4274, 4275, 4276, 4278, 4279, 4280, 4281, 4289, 4290, 4291, 4293, 4294, 4295, 4296, 42981, 42982, 42989, 4299, 4400, 4401, 4402, 4408, 4409, 4410, 4411, 4412, 4413, 4414, 4415, 4416, 4417, 4420, 4421, 4422, 4423, 4429, 4430, 4431, 4438, 4439, 4440, 4441, 4442, 4448, 4449, 4470, 4471, 4472, 4473, 4474, 4475, 4476, 4477, 4478, 4479, 4480, 4481, 4489, 4510, 4511, 4512, 4519, 4530, 4531, 4532, 4533, 4538, 4540, 4541, 4542, 4549, 4560, 4561, 4562, 4563, 4564, 4565, 4566, 4568, 4570, 4571, 4572, 4578, 4579, 4580, 4581, 4589, 4591, 4592, 4598, 4599, 7078, 7854, 9971, 9979, 9982 | I269, I260, I279, I278, I270, I271, I272, I281, I289, I280, I288, I301, I308, I309, I300, I319, I311, I313, I318, I312, I310, I330, I339, I340, I341, I342, I349, I348, I358, I359, I350, I351, I352, I360, I368, I362, I361, I369, I379, I371, I378, I372, I370, I38, I401, I408, I400, I409, I427, I425, I426, I428, I420, I421, I429, I424, I422, I423, I444, I445, I441, I442, I440, I447, I446, I443, I450, I456, I453, I454, I451, I458, I459, I455, I452, I469, I472, I470, I471, I479, I480, I481, I484, I483, I482, I489, I493, I494, I491, I498, I499, I492, I490, I495, I509, I501, I510, I512, I513, I519, I511, I518, I514, I515, I517, I7021, I7020, I7090, I7091, I711, I712, I713, I714, I715, I716, I718, I710, I719, I726, I723, I725, I721, I728, I724, I720, I722, I729, I730, I738, I731, I739, I742, I744, I745, I740, I743, I749, I741, I748, I771, I772, I773, I774, I775, I776, I770, I778, I779, I781, I780, I788, I789, I790, I791, I798, I803, I808, I800, I809, I801, I802, I81, I828, I829, I823, I820, I821, I822, I831, I830, I839, I832, I850, I861, I864, I860, I863, I862, I868, I878, I871, I872, I870, I879, I880, I881, I889, I888, I890, I891, I898, I899, I959, I958, I952, I951, I950, I972, I970, I971, I978, I99 |
| Cerebrovascular Disease | 4320, 4321, 4329, 4330, 4331, 4332, 4333, 4338, 4339, 4340, 4341, 4349, 4359 | I609, I619, I620, I629, I621, I630, I635, I632, I633, I631, I634, I652, I651, I658, I650, I659, I661, I669, I662, I660, I678, G458, G459, G450, G451 |
| Heart Failure | 4280, 4281, 4289 | I509, I501 |

**Supplementary Table 4:** Classification of T2D Prescriptions

| **LA Insulin** | MIXTARD, HUMALOG, HUMALOG, HUMALOG MIX, INSULATARD, HUMAN INSULATARD, INSULATARD GE, INSULATARD, HUMAN INSULATARD, INSULATARD PENFILL, MIXTARD, HUMAN MIXTARD, HUMULIN, HUMULIN I, HUMULIN, HUMULIN, INS HUMULIN, INS HUM, HUMULIN I, INSULATARD, INSULATARD PENFILL, INSULIN ASPART, BIP, ISOPHANE, BIPHASIC ISOPHANE, INSULIN BIPHASIC, ISOPHANE, INSULIN ISOPHANE, HUMULIN, INSULIN HUMULIN, MONOTARD, INSULIN GLARGINE, ISOPHANE, HUMULIN, HUMULIN I, INSULIN HUMULIN, LANTUS, NOVOMIX, INSULIN DEGLUDEC, LANTUS, LANTUS INJ |
| --- | --- |
| **SA Insulin** | ACTRAPID, ACTRAPID, ACTRAPID NOVOLET, HUMALOG, ACTRAPID, ACTRAPID PENFILL, HUMAN ACTRAPID, INSULATARD, HUMAN INSULATARD, INSULATARD, INSULATARD PENFILL, HUMAN INSULATARD, HUMULIN, HUMULIN, HUMULIN S, HUMULIN, INS HUMULIN, INS HUM, INSULATARD, INSULATARD PENFILL, INSULIN ASPART, HUMULIN, HUMULIN S, INSULIN HUMULIN, HUMULIN, INSULIN HUMULIN, INSULIN SOLUBLE, HUMALOG, HUMALOG INJ |
| **Sulfonylureas** | GLIBENCLAMIDE, GLICLAZIDE, DIAMICRON |
| **Biguanides** | METFORMIN, GLUCOPHAGE |
| **TZD** | ACTOS, PIOGLITAZONE, ROSIGLITAZONE, COMPETACT, AVANDAMET, AVANDIA, ROMOZIN |
| **GLP1** | XULTOPHY, BYETTA, BYDUREON, EXENATIDE, VICTOZA, LIRAGLUTIDE, LYXUMIA, LIXISENATIDE, DULAGLUTIDE, TRULICITY |
| **SGLT2** | EMPAGLIFLOZIN, FORXIGA, CANAGLIFLOZIN, DAPAGLIFLOZIN, NATEGLINIDE, NOVONORM, INVOKANA, STARLIX, REPAGLINIDE, JARDIANCE |
| **DPP4** | ALOGLIPTIN, VILDAGLIPTIN, JANUVIA, SITAGLIPTIN, JANUMET, LINAGLIPTIN, SAXAGLIPTIN, VILDAGLIPTIN, VILDAGLIPTIN/METFORMIN\, EUCREAS, GALVUS, VIPIDIA, ONGLYZA, TRAJENTA, VIPDOMET |
| **AGI** | ACARBOSE, GLUCOBAY |

**Supplementary Table 5:** The C4 association with time to cardiac event outcomes among individuals in the UKBiobank with T2D (N=23,061).

| **Cardiovascular Event** | **Number of events** | ***P* value** | **FDR *P*** | **HR** | **95% CI Low** | **95% CI High** |
| --- | --- | --- | --- | --- | --- | --- |
| MI | 2493 | 0.243 | 0.405 | 1.048 | 0.969 | 1.133 |
| stroke | 2363 | 0.052 | 0.26 | 1.108 | 0.999 | 1.23 |
| Heart failure | 537 | 0.338 | 0.423 | 0.955 | 0.869 | 1.05 |
| Cardiovascular disease | 3622 | 0.243 | 0.405 | 1.028 | 0.982 | 1.076 |
| Cerebrovascular disease | 875 | 0.74 | 0.74 | 0.982 | 0.883 | 1.092 |

**Supplementary Table 6:** C4 is not significantly associated with number of unique T2D medications.

| **Medication** | **Number of events** | ***P* value** | **OR** |
| --- | --- | --- | --- |
| Number of medications | 9,301 | 0.41 | 0.99 |

**Supplementary Table 7:** *IGF2R* and *MAS1* SNP associates with C4 subtype in UKBiobank cohort and CADD scores.

| rsid | Gene | Position | Allele | CADD | *P* value | OR |
| --- | --- | --- | --- | --- | --- | --- |
| rs2277070 | *IGF2R* | 160445793 | G | 0.00 | 0.075 | 0.989 |
| rs998203 | *IGF2R* | 160517888 | T | 0.01 | 0.027 | 1.01 |
| rs9457795 | *IGF2R* | 160395632 | G | 0.01 | 1.10E-41 | 1.063 |
| rs4709392 | *IGF2R* | 160460362 | A | 0.06 | 3.66E-08 | 1.025 |
| rs80254170 | *IGF2R* | 160514283 | G | 0.06 | 1.25E-06 | 0.962 |
| rs910173 | *MAS1* | 160333305 | G | 0.11 | 0.028 | 0.985 |
| rs2297372 | *IGF2R* | 160525287 | C | 0.16 | 0.032 | 1.01 |
| rs8191725 | *IGF2R* | 160429357 | G | 0.19 | 1.94E-17 | 1.102 |
| rs8191871 | *IGF2R* | 160490648 | T | 0.51 | 0.063 | 0.984 |
| rs8191774 | *IGF2R* | 160461793 | C | 0.58 | 0.051 | 1.03 |
| rs220721 | *MAS1* | 160328620 | T | 0.80 | 1.47E-70 | 1.094 |
| rs76667637 | *IGF2R* | 160423414 | G | 0.84 | 0.006 | 0.956 |
| rs170219 | *MAS1* | 160329423 | G | 1.50 | 1.63E-46 | 1.066 |
| rs7750288 | *IGF2R* | 160400147 | G | 1.75 | 5.62E-13 | 0.967 |
| rs1479355 | *IGF2R* | 160530626 | C | 2.17 | 0.250 | 1.006 |
| rs4709393 | *IGF2R* | 160472032 | G | 2.29 | 0.407 | 1.004 |
| rs4596512 | *IGF2R* | 160424901 | C | 2.36 | 2.17E-24 | 1.064 |
| rs635551 | *IGF2R* | 160404832 | C | 2.60 | 0.260 | 0.994 |
| rs77153348 | *IGF2R* | 160437880 | G | 4.01 | 0.0369 | 1.016 |
| rs78425119 | *IGF2R* | 160501825 | A | 4.88 | 1.21E-05 | 0.964 |
| rs6917747 | *IGF2R* | 160402705 | A | 5.09 | 1.53E-08 | 0.966 |
| rs78423775 | *IGF2R* | 160519228 | A | 5.38 | 0.720 | 0.997 |
| rs117727234 | *IGF2R* | 160533827 | A | 5.47 | 0.314 | 1.015 |
| rs879862 | *IGF2R* | 160398706 | T | 6.25 | 8.90E-15 | 1.041 |
| rs12207188 | *MAS1* | 160331654 | T | 6.35 | 0.065 | 0.984 |
| rs3777420 | *IGF2R* | 160394730 | G | 6.81 | 1.45E-29 | 1.069 |
| rs4709396 | *IGF2R* | 160497890 | T | 9.21 | 2.39E-17 | 1.091 |
| rs1805075 | *IGF2R* | 160505207 | G | 11.40 | 2.46E-15 | 1.082 |
| rs68168937 | *IGF2R* | 160451398 | T | 11.95 | 6.40E-14 | 1.04 |
| rs8191754 | *IGF2R* | 160448324 | G | 22.50 | 0.10107235 | 0.99 |
| rs3798186 | *IGF2R* | 160447017 | G | NA | 0.02829154 | 0.99 |
| rs614754 | *IGF2R* | 160505199 | C | NA | 0.01309584 | 0.884 |
| rs629849 | *IGF2R* | 160494409 | A | NA | 0.60958045 | 0.997 |
| rs642588 | *IGF2R* | 160411267 | A | NA | 3.81E-07 | 0.976 |
| rs220722 | *MAS1* | 160326690 | A | NA | 0.13061015 | 0.987 |
| rs220725 | *MAS1* | 160325989 | A | NA | 0.02021894 | 0.961 |
| rs220733 | *MAS1* | 160318803 | A | NA | 0.10916284 | 0.976 |

**Supplementary Table 8:** *MAS1* and *IGF2R* SNPs compared using logistic regression to test for associations with Type 2 Diabetes. Covariates that were included in the model are listed in row 2 and sample size is listed in row 3.

| **SNP** | **P-Value** | **FDR** | **OR** |
| --- | --- | --- | --- |
| *IGF2R*_rs1479355_C | 0.02 | 0.07 | 0.98 |
| *IGF2R*_rs117727234_A | 0.04 | 0.13 | 0.93 |
| *MAS1*_rs220721_T | 0.06 | 0.10 | 1.02 |
| *IGF2R*_rs80254170_G | 0.23 | 0.50 | 0.98 |
| *MAS1*_rs910173_G | 0.26 | 0.39 | 0.98 |
| *IGF2R*_rs2297372_C | 0.30 | 0.45 | 0.99 |
| *IGF2R*_rs1805075_G | 0.31 | 0.41 | 1.02 |
| *MAS1*_rs170219_G | 0.32 | 0.42 | 1.01 |
| *IGF2R*_rs8191725_G | 0.36 | 0.36 | 1.03 |
| *IGF2R*_rs629849_A | 0.37 | 0.56 | 1.01 |
| *IGF2R*_rs77153348_G | 0.39 | 0.59 | 0.98 |
| *IGF2R*_rs9457795_G | 0.40 | 0.61 | 1.01 |
| *IGF2R*_rs635551_C | 0.41 | 0.62 | 1.01 |
| *IGF2R*_rs4596512_C | 0.43 | 0.64 | 1.01 |
| *IGF2R*_rs642588_A | 0.46 | 0.55 | 0.99 |
| *IGF2R*_rs68168937_T | 0.48 | 0.57 | 1.01 |
| *IGF2R*_rs4709392_A | 0.49 | 0.68 | 1.01 |
| *IGF2R*_rs7750288_G | 0.50 | 0.61 | 0.99 |
| *IGF2R*_rs3777420_G | 0.51 | 0.77 | 1.01 |
| *MAS1*_rs220722_A | 0.52 | 0.57 | 0.99 |
| *IGF2R*_rs78423775_A | 0.54 | 0.99 | 0.99 |
| *IGF2R*_rs4709396_T | 0.54 | 0.54 | 1.01 |
| *IGF2R*_rs8191871_T | 0.57 | 0.57 | 1.01 |
| *MAS1*_rs12207188_T | 0.58 | 0.87 | 1.01 |
| *IGF2R*_rs78425119_A | 0.60 | 0.89 | 0.99 |
| *IGF2R*_rs76667637_G | 0.64 | 0.64 | 1.02 |
| *IGF2R*_rs8191774_C | 0.69 | 0.99 | 1.02 |
| *IGF2R*_rs614754_C | 0.71 | 0.87 | 1.04 |
| *MAS1*_rs220725_A | 0.71 | 0.97 | 0.99 |
| *IGF2R*_rs6917747_A | 0.72 | 0.72 | 1.00 |
| *IGF2R*_rs879862_T | 0.73 | 0.98 | 1.00 |
| *IGF2R*_rs998203_T | 0.80 | 0.95 | 1.00 |
| *IGF2R*_rs3798186_G | 0.81 | 0.98 | 1.00 |
| *IGF2R*_rs2277070_G | 0.84 | 0.84 | 1.00 |
| *IGF2R*_rs8191754_G | 0.87 | 0.87 | 1.00 |
| *IGF2R*_rs4709393_G | 0.87 | 0.87 | 1.00 |
| *MAS1*_rs220733_A | 0.97 | 0.97 | 1.00 |

**Supplementary Table 9:** *MAS1* and *IGF2R* SNPs compared using Cox proportional hazard model and linear regression to test for associations with age at T2D diagnosis, and HbA1c at appointment one. Covariates that were included in the model are listed in row 2 and sample size is listed in row 3.

|  | **HbA1c** | | | **Age at Diagnosis** | | |
| --- | --- | --- | --- | --- | --- | --- |
|  | **Adjusted for BMI, Age at Apt 1, and Sex** | | | **Adjusted for BMI, Sex** | | |
|  | **Cohort: T2D (24,580)** | | | **Cohort: T2D (24,580)** | | |
| SNP | pvalue | adjusted_p | Beta | pvalue | adjusted_p | HR |
| *IGF2R*_rs117727234_A | 0.98 | 0.98 | 0.00 | 0.10 | 0.16 | 0.94 |
| *IGF2R*_rs1479355_C | 0.76 | 0.76 | 0.00 | 0.06 | 0.09 | 0.98 |
| *IGF2R*_rs1805075_G | 0.41 | 0.41 | -0.02 | 0.06 | 0.19 | 1.04 |
| *IGF2R*_rs2277070_G | 0.22 | 0.66 | -0.02 | 0.51 | 0.76 | 0.99 |
| *IGF2R*_rs2297372_C | 0.45 | 0.45 | -0.01 | 0.35 | 0.45 | 0.99 |
| *IGF2R*_rs3777420_G | 0.91 | 0.91 | 0.00 | 0.48 | 0.77 | 1.01 |
| *IGF2R*_rs3798186_G | 0.02 | 0.07 | 0.03 | 0.98 | 0.98 | 1.00 |
| *IGF2R*_rs4596512_C | 0.77 | 0.77 | 0.01 | 0.37 | 0.64 | 1.01 |
| *IGF2R*_rs4709392_A | 0.63 | 0.68 | -0.01 | 0.68 | 0.68 | 1.00 |
| *IGF2R*_rs4709393_G | 0.53 | 0.87 | 0.01 | 0.67 | 0.87 | 1.00 |
| *IGF2R*_rs4709396_T | 0.32 | 0.48 | -0.03 | 0.16 | 0.47 | 1.03 |
| *IGF2R*_rs614754_C | 0.87 | 0.87 | 0.02 | 0.59 | 0.87 | 1.05 |
| *IGF2R*_rs629849_A | 0.34 | 0.56 | 0.02 | 0.56 | 0.56 | 1.01 |
| *IGF2R*_rs635551_C | 0.32 | 0.62 | 0.02 | 0.73 | 0.73 | 1.00 |
| *IGF2R*_rs642588_A | 0.55 | 0.55 | -0.01 | 0.26 | 0.55 | 0.99 |
| *IGF2R*_rs68168937_T | 0.57 | 0.57 | 0.01 | 0.37 | 0.57 | 1.01 |
| *IGF2R*_rs6917747_A | 0.62 | 0.72 | -0.01 | 0.57 | 0.72 | 0.99 |
| *IGF2R*_rs76667637_G | 0.26 | 0.64 | 0.05 | 0.44 | 0.64 | 1.03 |
| *IGF2R*_rs77153348_G | 0.69 | 0.69 | 0.01 | 0.36 | 0.59 | 0.98 |
| *IGF2R*_rs7750288_G | 0.61 | 0.61 | -0.01 | 0.31 | 0.61 | 0.99 |
| *IGF2R*_rs78423775_A | 0.99 | 0.99 | 0.00 | 0.68 | 0.99 | 0.99 |
| *IGF2R*_rs78425119_A | 0.27 | 0.81 | 0.03 | 0.89 | 0.89 | 1.00 |
| *IGF2R*_rs80254170_G | 0.33 | 0.50 | 0.02 | 0.59 | 0.59 | 0.99 |
| *IGF2R*_rs8191725_G | 0.23 | 0.36 | -0.04 | 0.25 | 0.36 | 1.03 |
| *IGF2R*_rs8191754_G | 0.26 | 0.78 | -0.02 | 0.53 | 0.80 | 0.99 |
| *IGF2R*_rs8191774_C | 0.99 | 0.99 | 0.00 | 0.59 | 0.99 | 1.02 |
| *IGF2R*_rs8191871_T | 0.30 | 0.57 | 0.02 | 0.50 | 0.57 | 1.01 |
| *IGF2R*_rs879862_T | 0.98 | 0.98 | 0.00 | 0.50 | 0.98 | 0.99 |
| *IGF2R*_rs9457795_G | 0.53 | 0.61 | -0.01 | 0.61 | 0.61 | 1.01 |
| *IGF2R*_rs998203_T | 0.66 | 0.95 | -0.01 | 0.95 | 0.95 | 1.00 |
| *MAS1*_rs12207188_T | 0.19 | 0.57 | 0.03 | 0.90 | 0.90 | 1.00 |
| *MAS1*_rs170219_G | 0.18 | 0.42 | -0.02 | 0.42 | 0.42 | 1.01 |
| *MAS1*_rs220721_T | 0.22 | 0.22 | -0.02 | 0.03 | 0.10 | 1.02 |
| *MAS1*_rs220722_A | 0.41 | 0.57 | 0.02 | 0.57 | 0.57 | 0.99 |
| *MAS1*_rs220725_A | 0.97 | 0.97 | 0.00 | 0.43 | 0.97 | 0.97 |
| *MAS1*_rs220733_A | 0.69 | 0.97 | 0.02 | 0.87 | 0.97 | 1.01 |
| *MAS1*_rs910173_G | 0.47 | 0.47 | -0.01 | 0.08 | 0.24 | 0.97 |

**Supplementary Table 10:** Association of *MAS1* and *IGF2R* SNPs with cardiac outcomes, among individuals with T2D (N=23,061).

| **Events** | **SNP** | **HR** | **95% CI Low** | **95% CI High** | ***P* Value** | **FDR *P* Value** |
| --- | --- | --- | --- | --- | --- | --- |
| Cardiovascular Disease (N=7603) | *MAS1*_rs220733_A | 1.154 | 1.026 | 1.299 | 0.017 | 0.085 |
|  | *MAS1*_rs220725_A | 1.036 | 0.904 | 1.186 | 0.613 | 0.863 |
|  | *MAS1*_rs220722_A | 1.042 | 0.975 | 1.113 | 0.226 | 0.68625 |
|  | *MAS1*_rs220721_T | 0.977 | 0.94 | 1.016 | 0.238 | 0.595 |
|  | *MAS1*_rs170219_G | 0.979 | 0.946 | 1.012 | 0.211 | 0.48333333 |
|  | *MAS1*_rs12207188_T | 1.063 | 0.993 | 1.138 | 0.077 | 0.385 |
|  | *MAS1*_rs910173_G | 1 | 0.948 | 1.055 | 0.996 | 0.996 |
|  | *IGF2R*_rs3777420_G | 0.984 | 0.94 | 1.03 | 0.483 | 0.817 |
|  | *IGF2R*_rs9457795_G | 0.988 | 0.954 | 1.022 | 0.482 | 0.857 |
|  | *IGF2R*_rs879862_T | 1.001 | 0.963 | 1.041 | 0.948 | 0.948 |
|  | *IGF2R*_rs7750288_G | 1.002 | 0.966 | 1.038 | 0.931 | 0.931 |
|  | *IGF2R*_rs6917747_A | 0.985 | 0.939 | 1.033 | 0.536 | 0.89333333 |
|  | *IGF2R*_rs635551_C | 1.068 | 1.022 | 1.116 | 0.004 | 0.02 |
|  | *IGF2R*_rs642588_A | 1.042 | 1.004 | 1.082 | 0.031 | 0.155 |
|  | *IGF2R*_rs76667637_G | 1.036 | 0.917 | 1.171 | 0.569 | 0.7325 |
|  | *IGF2R*_rs4596512_C | 0.959 | 0.916 | 1.004 | 0.072 | 0.36 |
|  | *IGF2R*_rs8191725_G | 1.066 | 0.975 | 1.166 | 0.159 | 0.3975 |
|  | *IGF2R*_rs77153348_G | 1.034 | 0.972 | 1.099 | 0.29 | 0.56 |
|  | *IGF2R*_rs2277070_G | 0.971 | 0.927 | 1.017 | 0.215 | 0.5575 |
|  | *IGF2R*_rs3798186_G | 1.014 | 0.98 | 1.049 | 0.427 | 0.85 |
|  | *IGF2R*_rs8191754_G | 0.975 | 0.931 | 1.021 | 0.278 | 0.695 |
|  | *IGF2R*_rs68168937_T | 0.987 | 0.948 | 1.028 | 0.526 | 0.703 |
|  | *IGF2R*_rs4709392_A | 0.986 | 0.953 | 1.02 | 0.42 | 0.55875 |
|  | *IGF2R*_rs8191774_C | 1.067 | 0.95 | 1.199 | 0.274 | 0.7225 |
|  | *IGF2R*_rs4709393_G | 0.974 | 0.939 | 1.01 | 0.157 | 0.3925 |
|  | *IGF2R*_rs8191871_T | 1.054 | 0.991 | 1.121 | 0.097 | 0.2425 |
|  | *IGF2R*_rs629849_A | 1.034 | 0.987 | 1.084 | 0.162 | 0.655 |
|  | *IGF2R*_rs4709396_T | 1.025 | 0.95 | 1.106 | 0.528 | 0.78625 |
|  | *IGF2R*_rs78425119_A | 0.976 | 0.912 | 1.045 | 0.487 | 0.81166667 |
|  | *IGF2R*_rs614754_C | 1.048 | 0.777 | 1.414 | 0.759 | 0.759 |
|  | *IGF2R*_rs1805075_G | 1.031 | 0.958 | 1.11 | 0.417 | 0.52125 |
|  | *IGF2R*_rs80254170_G | 0.94 | 0.881 | 1.003 | 0.063 | 0.2825 |
|  | *IGF2R*_rs998203_T | 1 | 0.968 | 1.034 | 0.983 | 0.983 |
|  | *IGF2R*_rs78423775_A | 1.064 | 0.999 | 1.134 | 0.055 | 0.1375 |
|  | *IGF2R*_rs2297372_C | 1.011 | 0.976 | 1.047 | 0.542 | 0.6775 |
|  | *IGF2R*_rs1479355_C | 1.025 | 0.988 | 1.064 | 0.195 | 0.46375 |
|  | *IGF2R*_rs117727234_A | 1.03 | 0.907 | 1.169 | 0.649 | 0.8925 |
| Cerebrovascular Disease (N=1435) | *MAS1*_rs220733_A | 0.994 | 0.746 | 1.324 | 0.968 | 0.968 |
|  | *MAS1*_rs220725_A | 0.96 | 0.693 | 1.331 | 0.808 | 0.863 |
|  | *MAS1*_rs220722_A | 0.95 | 0.81 | 1.113 | 0.522 | 0.68625 |
|  | *MAS1*_rs220721_T | 1.029 | 0.942 | 1.124 | 0.527 | 0.71875 |
|  | *MAS1*_rs170219_G | 1.043 | 0.966 | 1.127 | 0.284 | 0.48333333 |
|  | *MAS1*_rs12207188_T | 1.033 | 0.882 | 1.21 | 0.685 | 0.789 |
|  | *MAS1*_rs910173_G | 1.032 | 0.915 | 1.164 | 0.606 | 0.97625 |
|  | *IGF2R*_rs3777420_G | 1.071 | 0.966 | 1.187 | 0.193 | 0.817 |
|  | *IGF2R*_rs9457795_G | 1.025 | 0.947 | 1.109 | 0.546 | 0.857 |
|  | *IGF2R*_rs879862_T | 1.025 | 0.937 | 1.121 | 0.591 | 0.79375 |
|  | *IGF2R*_rs7750288_G | 0.951 | 0.875 | 1.034 | 0.239 | 0.39833333 |
|  | *IGF2R*_rs6917747_A | 0.941 | 0.843 | 1.051 | 0.283 | 0.7075 |
|  | *IGF2R*_rs635551_C | 0.985 | 0.888 | 1.094 | 0.781 | 0.821 |
|  | *IGF2R*_rs642588_A | 1.029 | 0.944 | 1.122 | 0.511 | 0.63875 |
|  | *IGF2R*_rs76667637_G | 0.973 | 0.729 | 1.3 | 0.855 | 0.855 |
|  | *IGF2R*_rs4596512_C | 1.009 | 0.91 | 1.119 | 0.864 | 0.986 |
|  | *IGF2R*_rs8191725_G | 1.039 | 0.844 | 1.28 | 0.716 | 0.895 |
|  | *IGF2R*_rs77153348_G | 0.945 | 0.816 | 1.094 | 0.448 | 0.56 |
|  | *IGF2R*_rs2277070_G | 0.968 | 0.871 | 1.076 | 0.548 | 0.779 |
|  | *IGF2R*_rs3798186_G | 1.005 | 0.93 | 1.087 | 0.895 | 0.895 |
|  | *IGF2R*_rs8191754_G | 0.975 | 0.877 | 1.085 | 0.643 | 0.80375 |
|  | *IGF2R*_rs68168937_T | 0.979 | 0.892 | 1.074 | 0.651 | 0.703 |
|  | *IGF2R*_rs4709392_A | 0.97 | 0.897 | 1.049 | 0.447 | 0.55875 |
|  | *IGF2R*_rs8191774_C | 1.064 | 0.815 | 1.39 | 0.648 | 0.78 |
|  | *IGF2R*_rs4709393_G | 0.998 | 0.918 | 1.086 | 0.966 | 0.966 |
|  | *IGF2R*_rs8191871_T | 0.947 | 0.817 | 1.097 | 0.468 | 0.468 |
|  | *IGF2R*_rs629849_A | 1.001 | 0.898 | 1.116 | 0.981 | 0.981 |
|  | *IGF2R*_rs4709396_T | 1.023 | 0.86 | 1.217 | 0.797 | 0.797 |
|  | *IGF2R*_rs78425119_A | 1.005 | 0.861 | 1.172 | 0.953 | 0.958 |
|  | *IGF2R*_rs614754_C | 1.388 | 0.762 | 2.528 | 0.284 | 0.47333333 |
|  | *IGF2R*_rs1805075_G | 1.007 | 0.85 | 1.193 | 0.932 | 0.932 |
|  | *IGF2R*_rs80254170_G | 0.953 | 0.82 | 1.107 | 0.526 | 0.6575 |
|  | *IGF2R*_rs998203_T | 1.062 | 0.986 | 1.145 | 0.114 | 0.52 |
|  | *IGF2R*_rs78423775_A | 1.12 | 0.971 | 1.292 | 0.12 | 0.2 |
|  | *IGF2R*_rs2297372_C | 1.032 | 0.952 | 1.117 | 0.446 | 0.6775 |
|  | *IGF2R*_rs1479355_C | 1.043 | 0.958 | 1.135 | 0.334 | 0.46375 |
|  | *IGF2R*_rs117727234_A | 1.171 | 0.889 | 1.543 | 0.26 | 0.7575 |
| Heart Failure (N=1829) | *MAS1*_rs220733_A | 1.123 | 0.884 | 1.428 | 0.342 | 0.855 |
|  | *MAS1*_rs220725_A | 0.921 | 0.685 | 1.237 | 0.583 | 0.863 |
|  | *MAS1*_rs220722_A | 1.042 | 0.911 | 1.192 | 0.549 | 0.68625 |
|  | *MAS1*_rs220721_T | 0.977 | 0.903 | 1.058 | 0.575 | 0.71875 |
|  | *MAS1*_rs170219_G | 0.992 | 0.926 | 1.064 | 0.831 | 0.831 |
|  | *MAS1*_rs12207188_T | 1.067 | 0.929 | 1.226 | 0.357 | 0.789 |
|  | *MAS1*_rs910173_G | 1.015 | 0.912 | 1.131 | 0.781 | 0.97625 |
|  | *IGF2R*_rs3777420_G | 0.989 | 0.901 | 1.086 | 0.817 | 0.817 |
|  | *IGF2R*_rs9457795_G | 1.006 | 0.938 | 1.08 | 0.857 | 0.857 |
|  | *IGF2R*_rs879862_T | 1.02 | 0.941 | 1.104 | 0.635 | 0.79375 |
|  | *IGF2R*_rs7750288_G | 1.067 | 0.993 | 1.148 | 0.078 | 0.195 |
|  | *IGF2R*_rs6917747_A | 1.071 | 0.974 | 1.177 | 0.156 | 0.7075 |
|  | *IGF2R*_rs635551_C | 1.011 | 0.922 | 1.108 | 0.821 | 0.821 |
|  | *IGF2R*_rs642588_A | 1.034 | 0.958 | 1.117 | 0.385 | 0.63875 |
|  | *IGF2R*_rs76667637_G | 1.082 | 0.846 | 1.384 | 0.528 | 0.7325 |
|  | *IGF2R*_rs4596512_C | 1.001 | 0.912 | 1.098 | 0.986 | 0.986 |
|  | *IGF2R*_rs8191725_G | 1.141 | 0.957 | 1.36 | 0.141 | 0.3975 |
|  | *IGF2R*_rs77153348_G | 0.948 | 0.833 | 1.079 | 0.421 | 0.56 |
|  | *IGF2R*_rs2277070_G | 1.013 | 0.923 | 1.113 | 0.779 | 0.779 |
|  | *IGF2R*_rs3798186_G | 0.982 | 0.916 | 1.053 | 0.611 | 0.85 |
|  | *IGF2R*_rs8191754_G | 1.009 | 0.919 | 1.109 | 0.847 | 0.847 |
|  | *IGF2R*_rs68168937_T | 1.016 | 0.936 | 1.102 | 0.703 | 0.703 |
|  | *IGF2R*_rs4709392_A | 1.02 | 0.952 | 1.093 | 0.578 | 0.578 |
|  | *IGF2R*_rs8191774_C | 1.035 | 0.815 | 1.314 | 0.78 | 0.78 |
|  | *IGF2R*_rs4709393_G | 1.003 | 0.931 | 1.081 | 0.933 | 0.966 |
|  | *IGF2R*_rs8191871_T | 1.059 | 0.933 | 1.202 | 0.375 | 0.468 |
|  | *IGF2R*_rs629849_A | 1.042 | 0.948 | 1.146 | 0.393 | 0.655 |
|  | *IGF2R*_rs4709396_T | 1.107 | 0.952 | 1.286 | 0.186 | 0.465 |
|  | *IGF2R*_rs78425119_A | 0.944 | 0.822 | 1.085 | 0.418 | 0.81166667 |
|  | *IGF2R*_rs614754_C | 0.611 | 0.295 | 1.265 | 0.184 | 0.47333333 |
|  | *IGF2R*_rs1805075_G | 1.098 | 0.949 | 1.27 | 0.208 | 0.52 |
|  | *IGF2R*_rs80254170_G | 0.951 | 0.833 | 1.085 | 0.453 | 0.6575 |
|  | *IGF2R*_rs998203_T | 1.014 | 0.948 | 1.084 | 0.695 | 0.983 |
|  | *IGF2R*_rs78423775_A | 0.915 | 0.798 | 1.049 | 0.203 | 0.25375 |
|  | *IGF2R*_rs2297372_C | 1.025 | 0.954 | 1.1 | 0.503 | 0.6775 |
|  | *IGF2R*_rs1479355_C | 1.035 | 0.96 | 1.117 | 0.371 | 0.46375 |
|  | *IGF2R*_rs117727234_A | 1.137 | 0.89 | 1.452 | 0.303 | 0.7575 |
| MI (N=2630) | *MAS1*_rs220733_A | 1.008 | 0.817 | 1.245 | 0.938 | 0.968 |
|  | *MAS1*_rs220725_A | 0.71 | 0.538 | 0.938 | 0.016 | 0.08 |
|  | *MAS1*_rs220722_A | 0.997 | 0.889 | 1.118 | 0.962 | 0.962 |
|  | *MAS1*_rs220721_T | 0.941 | 0.88 | 1.007 | 0.077 | 0.385 |
|  | *MAS1*_rs170219_G | 0.993 | 0.937 | 1.052 | 0.806 | 0.831 |
|  | *MAS1*_rs12207188_T | 1.032 | 0.918 | 1.16 | 0.596 | 0.789 |
|  | *MAS1*_rs910173_G | 1.1 | 1.008 | 1.2 | 0.033 | 0.165 |
|  | *IGF2R*_rs3777420_G | 0.984 | 0.91 | 1.064 | 0.689 | 0.817 |
|  | *IGF2R*_rs9457795_G | 0.994 | 0.937 | 1.054 | 0.839 | 0.857 |
|  | *IGF2R*_rs879862_T | 1.038 | 0.971 | 1.109 | 0.27 | 0.79375 |
|  | *IGF2R*_rs7750288_G | 1.059 | 0.997 | 1.125 | 0.065 | 0.195 |
|  | *IGF2R*_rs6917747_A | 1.009 | 0.931 | 1.093 | 0.833 | 0.934 |
|  | *IGF2R*_rs635551_C | 1.014 | 0.94 | 1.094 | 0.717 | 0.821 |
|  | *IGF2R*_rs642588_A | 1.006 | 0.944 | 1.073 | 0.855 | 0.855 |
|  | *IGF2R*_rs76667637_G | 1.194 | 0.982 | 1.45 | 0.075 | 0.375 |
|  | *IGF2R*_rs4596512_C | 0.97 | 0.898 | 1.049 | 0.45 | 0.986 |
|  | *IGF2R*_rs8191725_G | 1.041 | 0.894 | 1.211 | 0.606 | 0.895 |
|  | *IGF2R*_rs77153348_G | 0.931 | 0.835 | 1.038 | 0.196 | 0.56 |
|  | *IGF2R*_rs2277070_G | 1.049 | 0.971 | 1.132 | 0.223 | 0.5575 |
|  | *IGF2R*_rs3798186_G | 0.988 | 0.932 | 1.047 | 0.68 | 0.85 |
|  | *IGF2R*_rs8191754_G | 1.06 | 0.982 | 1.145 | 0.138 | 0.69 |
|  | *IGF2R*_rs68168937_T | 1.053 | 0.985 | 1.127 | 0.129 | 0.3225 |
|  | *IGF2R*_rs4709392_A | 1.061 | 1.002 | 1.123 | 0.044 | 0.22 |
|  | *IGF2R*_rs8191774_C | 1.029 | 0.843 | 1.257 | 0.778 | 0.78 |
|  | *IGF2R*_rs4709393_G | 0.992 | 0.932 | 1.056 | 0.796 | 0.966 |
|  | *IGF2R*_rs8191871_T | 1.15 | 1.039 | 1.273 | 0.007 | 0.035 |
|  | *IGF2R*_rs629849_A | 1.041 | 0.961 | 1.126 | 0.325 | 0.655 |
|  | *IGF2R*_rs4709396_T | 1.107 | 0.978 | 1.254 | 0.108 | 0.465 |
|  | *IGF2R*_rs78425119_A | 0.909 | 0.807 | 1.023 | 0.114 | 0.57 |
|  | *IGF2R*_rs614754_C | 0.848 | 0.477 | 1.505 | 0.572 | 0.715 |
|  | *IGF2R*_rs1805075_G | 1.092 | 0.967 | 1.232 | 0.155 | 0.52 |
|  | *IGF2R*_rs80254170_G | 0.913 | 0.816 | 1.022 | 0.113 | 0.2825 |
|  | *IGF2R*_rs998203_T | 0.999 | 0.945 | 1.056 | 0.973 | 0.983 |
|  | *IGF2R*_rs78423775_A | 0.96 | 0.858 | 1.075 | 0.48 | 0.48 |
|  | *IGF2R*_rs2297372_C | 0.988 | 0.93 | 1.048 | 0.683 | 0.683 |
|  | *IGF2R*_rs1479355_C | 0.987 | 0.926 | 1.052 | 0.678 | 0.678 |
|  | *IGF2R*_rs117727234_A | 1.006 | 0.81 | 1.249 | 0.959 | 0.959 |
| Stroke (N=1490) | *MAS1*_rs220733_A | 0.925 | 0.69 | 1.24 | 0.602 | 0.968 |
|  | *MAS1*_rs220725_A | 0.972 | 0.707 | 1.337 | 0.863 | 0.863 |
|  | *MAS1*_rs220722_A | 0.942 | 0.805 | 1.102 | 0.455 | 0.68625 |
|  | *MAS1*_rs220721_T | 1.009 | 0.925 | 1.102 | 0.833 | 0.833 |
|  | *MAS1*_rs170219_G | 1.042 | 0.965 | 1.125 | 0.29 | 0.48333333 |
|  | *MAS1*_rs12207188_T | 1.022 | 0.874 | 1.194 | 0.789 | 0.789 |
|  | *MAS1*_rs910173_G | 1.076 | 0.958 | 1.21 | 0.216 | 0.54 |
|  | *IGF2R*_rs3777420_G | 1.014 | 0.915 | 1.124 | 0.792 | 0.817 |
|  | *IGF2R*_rs9457795_G | 1.039 | 0.962 | 1.123 | 0.33 | 0.857 |
|  | *IGF2R*_rs879862_T | 1.038 | 0.951 | 1.134 | 0.404 | 0.79375 |
|  | *IGF2R*_rs7750288_G | 1.014 | 0.935 | 1.099 | 0.744 | 0.93 |
|  | *IGF2R*_rs6917747_A | 1.005 | 0.903 | 1.118 | 0.934 | 0.934 |
|  | *IGF2R*_rs635551_C | 0.969 | 0.874 | 1.074 | 0.548 | 0.821 |
|  | *IGF2R*_rs642588_A | 1.058 | 0.972 | 1.151 | 0.191 | 0.4775 |
|  | *IGF2R*_rs76667637_G | 0.922 | 0.688 | 1.235 | 0.586 | 0.7325 |
|  | *IGF2R*_rs4596512_C | 0.988 | 0.892 | 1.095 | 0.823 | 0.986 |
|  | *IGF2R*_rs8191725_G | 0.989 | 0.803 | 1.218 | 0.918 | 0.918 |
|  | *IGF2R*_rs77153348_G | 0.965 | 0.837 | 1.113 | 0.628 | 0.628 |
|  | *IGF2R*_rs2277070_G | 1.024 | 0.924 | 1.135 | 0.651 | 0.779 |
|  | *IGF2R*_rs3798186_G | 0.972 | 0.9 | 1.05 | 0.476 | 0.85 |
|  | *IGF2R*_rs8191754_G | 1.035 | 0.933 | 1.147 | 0.516 | 0.80375 |
|  | *IGF2R*_rs68168937_T | 0.926 | 0.844 | 1.016 | 0.105 | 0.3225 |
|  | *IGF2R*_rs4709392_A | 0.94 | 0.87 | 1.016 | 0.119 | 0.2975 |
|  | *IGF2R*_rs8191774_C | 0.854 | 0.639 | 1.143 | 0.289 | 0.7225 |
|  | *IGF2R*_rs4709393_G | 0.933 | 0.858 | 1.015 | 0.106 | 0.3925 |
|  | *IGF2R*_rs8191871_T | 0.937 | 0.809 | 1.084 | 0.382 | 0.468 |
|  | *IGF2R*_rs629849_A | 1.016 | 0.913 | 1.13 | 0.77 | 0.9625 |
|  | *IGF2R*_rs4709396_T | 1.043 | 0.88 | 1.235 | 0.629 | 0.78625 |
|  | *IGF2R*_rs78425119_A | 1.004 | 0.863 | 1.169 | 0.958 | 0.958 |
|  | *IGF2R*_rs614754_C | 0.551 | 0.21 | 1.445 | 0.226 | 0.47333333 |
|  | *IGF2R*_rs1805075_G | 1.07 | 0.91 | 1.258 | 0.413 | 0.52125 |
|  | *IGF2R*_rs80254170_G | 0.985 | 0.852 | 1.14 | 0.842 | 0.842 |
|  | *IGF2R*_rs998203_T | 1.049 | 0.974 | 1.129 | 0.208 | 0.52 |
|  | *IGF2R*_rs78423775_A | 1.17 | 1.019 | 1.343 | 0.026 | 0.13 |
|  | *IGF2R*_rs2297372_C | 1.045 | 0.966 | 1.13 | 0.273 | 0.6775 |
|  | *IGF2R*_rs1479355_C | 1.07 | 0.985 | 1.163 | 0.111 | 0.46375 |
|  | *IGF2R*_rs117727234_A | 1.054 | 0.796 | 1.396 | 0.714 | 0.8925 |

|  |  |  |
| --- | --- | --- |

**Supplementary Table 11:** Association of *MAS1* and *IGF2R* SNPs with the number of unique T2D medications.

|  | **# T2D Prescriptions** (*N*=9,301) | |
| --- | --- | --- |
| **SNP** | **OR** | **P Value** |
| *MAS1*_rs220733_A | 1.041 | 0.347 |
| *MAS1*_rs220725_A | 1.007 | 0.475 |
| *MAS1*_rs220722_A | 1.011 | 0.421 |
| *MAS1*_rs220721_T | 0.944 | 0.038 |
| *MAS1*_rs170219_G | 0.948 | 0.032 |
| *MAS1*_rs12207188_T | 1.034 | 0.282 |
| *MAS1*_rs910173_G | 0.993 | 0.438 |
| *IGF2R*_rs3777420_G | 0.938 | 0.047 |
| *IGF2R*_rs9457795_G | 0.959 | 0.071 |
| *IGF2R*_rs879862_T | 0.973 | 0.203 |
| *IGF2R*_rs7750288_G | 0.974 | 0.185 |
| *IGF2R*_rs6917747_A | 0.961 | 0.155 |
| *IGF2R*_rs635551_C | 1.022 | 0.284 |
| *IGF2R*_rs642588_A | 1.017 | 0.292 |
| *IGF2R*_rs76667637_G | 1.071 | 0.253 |
| *IGF2R*_rs4596512_C | 1.01 | 0.4 |
| *IGF2R*_rs8191725_G | 0.975 | 0.371 |
| *IGF2R*_rs77153348_G | 0.957 | 0.196 |
| *IGF2R*_rs2277070_G | 1.028 | 0.233 |
| *IGF2R*_rs3798186_G | 1.005 | 0.427 |
| *IGF2R*_rs8191754_G | 1.022 | 0.279 |
| *IGF2R*_rs68168937_T | 1.007 | 0.415 |
| *IGF2R*_rs4709392_A | 1.005 | 0.433 |
| *IGF2R*_rs8191774_C | 1.01 | 0.458 |
| *IGF2R*_rs4709393_G | 0.983 | 0.286 |
| *IGF2R*_rs8191871_T | 1.008 | 0.438 |
| *IGF2R*_rs629849_A | 1.04 | 0.159 |
| *IGF2R*_rs4709396_T | 1.039 | 0.273 |
| *IGF2R*_rs78425119_A | 1.002 | 0.487 |
| *IGF2R*_rs614754_C | 0.682 | 0.088 |
| *IGF2R*_rs1805075_G | 1.056 | 0.187 |
| *IGF2R*_rs80254170_G | 1.003 | 0.478 |
| *IGF2R*_rs998203_T | 1.059 | 0.019 |
| *IGF2R*_rs78423775_A | 1.098 | 0.041 |
| *IGF2R*_rs2297372_C | 1.073 | 0.008 |
| *IGF2R*_rs1479355_C | 1.08 | 0.007 |
| *IGF2R*_rs117727234_A | 0.811 | 0.027 |

**Supplementary Table 12:** *MAS1* and *IGF2R* SNPs associations with T2D prescription classes (N=10,990).

| SNP | Medication | P Value | FDR P Value | OR | 95% CI Low | 95% CI High |
| --- | --- | --- | --- | --- | --- | --- |
| IGF2R_rs117727234_A | AGI (N=141) | 0.279 | 0.372 | 0.463 | 0.077 | 1.445 |
|  | biguanides (N=8411) | 0.447 | 0.457 | 0.907 | 0.709 | 1.171 |
|  | DPP4 (N=2509) | 0.279 | 0.372 | 0.864 | 0.658 | 1.119 |
|  | GLP1 (N=902) | 0.457 | 0.457 | 0.854 | 0.552 | 1.269 |
|  | insulin (N=3126) | 0.01 | 0.082 | 0.717 | 0.552 | 0.919 |
|  | SGLT2 (N=734) | 0.109 | 0.29 | 0.661 | 0.382 | 1.059 |
|  | sulfonylureas (N=5653) | 0.021 | 0.085 | 0.777 | 0.627 | 0.962 |
|  | TZD (N=2295) | 0.148 | 0.297 | 0.814 | 0.611 | 1.068 |
|  | summary (N=NA) | 0.238 | NA | 0.821 | 0.592 | 1.139 |
| IGF2R_rs1479355_C | AGI (N=141) | 0.324 | 0.432 | 1.146 | 0.869 | 1.495 |
|  | biguanides (N=8411) | 0.189 | 0.378 | 0.949 | 0.878 | 1.026 |
|  | DPP4 (N=2509) | 0.38 | 0.434 | 1.034 | 0.959 | 1.114 |
|  | GLP1 (N=902) | 0.066 | 0.378 | 0.891 | 0.787 | 1.007 |
|  | insulin (N=3126) | 0.873 | 0.873 | 0.994 | 0.927 | 1.066 |
|  | SGLT2 (N=734) | 0.101 | 0.378 | 1.109 | 0.979 | 1.255 |
|  | sulfonylureas (N=5653) | 0.159 | 0.378 | 1.047 | 0.982 | 1.116 |
|  | TZD (N=2295) | 0.238 | 0.381 | 1.048 | 0.969 | 1.133 |
|  | summary (N=NA) | 0.995 | NA | 0.999 | 0.835 | 1.196 |
| IGF2R_rs1805075_G | AGI (N=141) | 0.579 | 0.926 | 0.848 | 0.448 | 1.45 |
|  | biguanides (N=8411) | 0.438 | 0.926 | 0.942 | 0.812 | 1.097 |
|  | DPP4 (N=2509) | 0.917 | 0.981 | 0.992 | 0.856 | 1.146 |
|  | GLP1 (N=902) | 0.944 | 0.981 | 0.991 | 0.774 | 1.253 |
|  | insulin (N=3126) | 0.554 | 0.926 | 1.041 | 0.909 | 1.19 |
|  | SGLT2 (N=734) | 0.059 | 0.472 | 1.242 | 0.985 | 1.547 |
|  | sulfonylureas (N=5653) | 0.981 | 0.981 | 0.999 | 0.881 | 1.131 |
|  | TZD (N=2295) | 0.231 | 0.924 | 1.095 | 0.942 | 1.269 |
|  | summary (N=NA) | 0.983 | NA | 0.997 | 0.777 | 1.28 |
| IGF2R_rs2277070_G | AGI (N=141) | 0.362 | 0.505 | 1.161 | 0.833 | 1.584 |
|  | biguanides (N=8411) | 0.007 | 0.052 | 1.143 | 1.039 | 1.26 |
|  | DPP4 (N=2509) | 0.123 | 0.328 | 1.073 | 0.981 | 1.174 |
|  | GLP1 (N=902) | 0.568 | 0.65 | 1.044 | 0.9 | 1.206 |
|  | insulin (N=3126) | 0.676 | 0.676 | 1.018 | 0.935 | 1.108 |
|  | SGLT2 (N=734) | 0.379 | 0.505 | 0.932 | 0.795 | 1.088 |
|  | sulfonylureas (N=5653) | 0.264 | 0.505 | 1.045 | 0.967 | 1.13 |
|  | TZD (N=2295) | 0.017 | 0.069 | 1.12 | 1.02 | 1.229 |
|  | summary (N=NA) | 0.436 | NA | 1.082 | 0.887 | 1.32 |
| IGF2R_rs2297372_C | AGI (N=141) | 0.227 | 0.376 | 1.17 | 0.903 | 1.506 |
|  | biguanides (N=8411) | 0.185 | 0.376 | 0.952 | 0.884 | 1.024 |
|  | DPP4 (N=2509) | 0.842 | 0.842 | 1.007 | 0.938 | 1.081 |
|  | GLP1 (N=902) | 0.235 | 0.376 | 0.933 | 0.832 | 1.045 |
|  | insulin (N=3126) | 0.645 | 0.813 | 1.016 | 0.951 | 1.084 |
|  | SGLT2 (N=734) | 0.711 | 0.813 | 1.023 | 0.908 | 1.15 |
|  | sulfonylureas (N=5653) | 0.083 | 0.376 | 1.055 | 0.993 | 1.12 |
|  | TZD (N=2295) | 0.17 | 0.376 | 1.052 | 0.978 | 1.132 |
|  | summary (N=NA) | 0.984 | NA | 1.002 | 0.842 | 1.192 |
| IGF2R_rs3777420_G | AGI (N=141) | 0.33 | 0.459 | 0.833 | 0.567 | 1.185 |
|  | biguanides (N=8411) | 0.021 | 0.083 | 0.895 | 0.816 | 0.984 |
|  | DPP4 (N=2509) | 0.159 | 0.425 | 0.936 | 0.852 | 1.026 |
|  | GLP1 (N=902) | 0.52 | 0.594 | 0.953 | 0.821 | 1.102 |
|  | insulin (N=3126) | 0.263 | 0.459 | 0.952 | 0.874 | 1.037 |
|  | SGLT2 (N=734) | 0.344 | 0.459 | 1.075 | 0.923 | 1.247 |
|  | sulfonylureas (N=5653) | 0.02 | 0.083 | 0.912 | 0.843 | 0.985 |
|  | TZD (N=2295) | 0.941 | 0.941 | 1.004 | 0.912 | 1.103 |
|  | summary (N=NA) | 0.457 | NA | 0.928 | 0.762 | 1.13 |
| IGF2R_rs3798186_G | AGI (N=141) | 0.442 | 0.722 | 0.904 | 0.697 | 1.164 |
|  | biguanides (N=8411) | 0.778 | 0.807 | 1.01 | 0.942 | 1.084 |
|  | DPP4 (N=2509) | 0.807 | 0.807 | 1.008 | 0.942 | 1.079 |
|  | GLP1 (N=902) | 0.451 | 0.722 | 0.959 | 0.859 | 1.069 |
|  | insulin (N=3126) | 0.355 | 0.722 | 0.971 | 0.912 | 1.034 |
|  | SGLT2 (N=734) | 0.562 | 0.75 | 0.967 | 0.862 | 1.083 |
|  | sulfonylureas (N=5653) | 0.262 | 0.722 | 1.033 | 0.976 | 1.095 |
|  | TZD (N=2295) | 0.182 | 0.722 | 1.049 | 0.978 | 1.125 |
|  | summary (N=NA) | 0.91 | NA | 1.01 | 0.852 | 1.197 |
| IGF2R_rs4596512_C | AGI (N=141) | 0.194 | 0.988 | 0.777 | 0.521 | 1.119 |
|  | biguanides (N=8411) | 0.545 | 0.988 | 0.971 | 0.883 | 1.069 |
|  | DPP4 (N=2509) | 0.43 | 0.988 | 1.037 | 0.946 | 1.136 |
|  | GLP1 (N=902) | 0.988 | 0.988 | 0.999 | 0.86 | 1.156 |
|  | insulin (N=3126) | 0.985 | 0.988 | 1.001 | 0.919 | 1.089 |
|  | SGLT2 (N=734) | 0.366 | 0.988 | 1.072 | 0.92 | 1.244 |
|  | sulfonylureas (N=5653) | 0.891 | 0.988 | 0.995 | 0.92 | 1.076 |
|  | TZD (N=2295) | 0.947 | 0.988 | 1.003 | 0.911 | 1.104 |
|  | summary (N=NA) | 0.946 | NA | 0.993 | 0.814 | 1.212 |
| IGF2R_rs4709392_A | AGI (N=141) | 0.474 | 0.941 | 1.095 | 0.851 | 1.4 |
|  | biguanides (N=8411) | 0.69 | 0.941 | 1.015 | 0.945 | 1.089 |
|  | DPP4 (N=2509) | 0.747 | 0.941 | 0.989 | 0.924 | 1.058 |
|  | GLP1 (N=902) | 0.405 | 0.941 | 1.047 | 0.939 | 1.167 |
|  | insulin (N=3126) | 0.897 | 0.941 | 0.996 | 0.935 | 1.061 |
|  | SGLT2 (N=734) | 0.356 | 0.941 | 0.947 | 0.843 | 1.062 |
|  | sulfonylureas (N=5653) | 0.941 | 0.941 | 0.998 | 0.942 | 1.057 |
|  | TZD (N=2295) | 0.857 | 0.941 | 1.007 | 0.938 | 1.08 |
|  | summary (N=NA) | 0.963 | NA | 1.004 | 0.846 | 1.191 |
| IGF2R_rs4709393_G | AGI (N=141) | 0.157 | 0.937 | 0.813 | 0.605 | 1.076 |
|  | biguanides (N=8411) | 0.758 | 0.937 | 0.988 | 0.916 | 1.066 |
|  | DPP4 (N=2509) | 0.886 | 0.937 | 1.005 | 0.935 | 1.081 |
|  | GLP1 (N=902) | 0.586 | 0.937 | 1.033 | 0.919 | 1.159 |
|  | insulin (N=3126) | 0.395 | 0.937 | 0.971 | 0.907 | 1.039 |
|  | SGLT2 (N=734) | 0.593 | 0.937 | 0.967 | 0.854 | 1.093 |
|  | sulfonylureas (N=5653) | 0.336 | 0.937 | 0.97 | 0.912 | 1.032 |
|  | TZD (N=2295) | 0.937 | 0.937 | 1.003 | 0.93 | 1.082 |
|  | summary (N=NA) | 0.864 | NA | 0.985 | 0.825 | 1.175 |
| IGF2R_rs4709396_T | AGI (N=141) | 0.676 | 0.928 | 0.882 | 0.463 | 1.519 |
|  | biguanides (N=8411) | 0.395 | 0.928 | 0.935 | 0.801 | 1.094 |
|  | DPP4 (N=2509) | 0.863 | 0.928 | 0.987 | 0.847 | 1.145 |
|  | GLP1 (N=902) | 0.928 | 0.928 | 0.989 | 0.766 | 1.259 |
|  | insulin (N=3126) | 0.716 | 0.928 | 1.026 | 0.892 | 1.178 |
|  | SGLT2 (N=734) | 0.186 | 0.928 | 1.175 | 0.919 | 1.482 |
|  | sulfonylureas (N=5653) | 0.765 | 0.928 | 0.981 | 0.862 | 1.116 |
|  | TZD (N=2295) | 0.264 | 0.928 | 1.092 | 0.934 | 1.271 |
|  | summary (N=NA) | 0.912 | NA | 0.986 | 0.765 | 1.271 |
| IGF2R_rs614754_C | AGI (N=141) | 0.988 | 0.995 | 0 | NA | 7.27E+20 |
|  | biguanides (N=8411) | 0.917 | 0.995 | 1.043 | 0.495 | 2.476 |
|  | DPP4 (N=2509) | 0.667 | 0.995 | 0.851 | 0.384 | 1.696 |
|  | GLP1 (N=902) | 0.192 | 0.995 | 0.375 | 0.059 | 1.287 |
|  | insulin (N=3126) | 0.995 | 0.995 | 0.998 | 0.505 | 1.861 |
|  | SGLT2 (N=734) | 0.529 | 0.995 | 0.63 | 0.101 | 2.096 |
|  | sulfonylureas (N=5653) | 0.296 | 0.995 | 0.728 | 0.397 | 1.32 |
|  | TZD (N=2295) | 0.378 | 0.995 | 0.709 | 0.307 | 1.448 |
|  | summary (N=NA) | 0.473 | NA | 0.782 | 0.4 | 1.53 |
| IGF2R_rs629849_A | AGI (N=141) | 0.164 | 0.521 | 1.263 | 0.897 | 1.737 |
|  | biguanides (N=8411) | 0.109 | 0.521 | 1.084 | 0.983 | 1.198 |
|  | DPP4 (N=2509) | 0.641 | 0.641 | 1.023 | 0.929 | 1.125 |
|  | GLP1 (N=902) | 0.591 | 0.641 | 0.958 | 0.819 | 1.117 |
|  | insulin (N=3126) | 0.195 | 0.521 | 1.06 | 0.97 | 1.157 |
|  | SGLT2 (N=734) | 0.4 | 0.641 | 1.071 | 0.911 | 1.252 |
|  | sulfonylureas (N=5653) | 0.6 | 0.641 | 1.022 | 0.942 | 1.108 |
|  | TZD (N=2295) | 0.535 | 0.641 | 1.032 | 0.934 | 1.138 |
|  | summary (N=NA) | 0.637 | NA | 1.05 | 0.858 | 1.286 |
| IGF2R_rs635551_C | AGI (N=141) | 0.36 | 0.93 | 0.847 | 0.584 | 1.193 |
|  | biguanides (N=8411) | 0.709 | 0.93 | 0.982 | 0.895 | 1.079 |
|  | DPP4 (N=2509) | 0.644 | 0.93 | 0.979 | 0.893 | 1.071 |
|  | GLP1 (N=902) | 0.365 | 0.93 | 0.935 | 0.808 | 1.079 |
|  | insulin (N=3126) | 0.917 | 0.93 | 0.996 | 0.915 | 1.083 |
|  | SGLT2 (N=734) | 0.295 | 0.93 | 0.92 | 0.785 | 1.073 |
|  | sulfonylureas (N=5653) | 0.93 | 0.93 | 1.003 | 0.929 | 1.083 |
|  | TZD (N=2295) | 0.533 | 0.93 | 1.03 | 0.938 | 1.13 |
|  | summary (N=NA) | 0.908 | NA | 0.989 | 0.812 | 1.203 |
| IGF2R_rs642588_A | AGI (N=141) | 0.138 | 0.553 | 1.226 | 0.931 | 1.597 |
|  | biguanides (N=8411) | 0.044 | 0.349 | 0.922 | 0.853 | 0.998 |
|  | DPP4 (N=2509) | 0.497 | 0.796 | 1.027 | 0.951 | 1.108 |
|  | GLP1 (N=902) | 0.416 | 0.796 | 0.951 | 0.841 | 1.073 |
|  | insulin (N=3126) | 0.725 | 0.829 | 0.987 | 0.919 | 1.06 |
|  | SGLT2 (N=734) | 0.623 | 0.829 | 1.032 | 0.908 | 1.171 |
|  | sulfonylureas (N=5653) | 0.969 | 0.969 | 1.001 | 0.939 | 1.068 |
|  | TZD (N=2295) | 0.384 | 0.796 | 0.965 | 0.891 | 1.045 |
|  | summary (N=NA) | 0.742 | NA | 0.97 | 0.81 | 1.162 |
| IGF2R_rs68168937_T | AGI (N=141) | 0.949 | 0.949 | 0.99 | 0.725 | 1.328 |
|  | biguanides (N=8411) | 0.165 | 0.746 | 0.943 | 0.868 | 1.025 |
|  | DPP4 (N=2509) | 0.216 | 0.746 | 0.95 | 0.876 | 1.03 |
|  | GLP1 (N=902) | 0.38 | 0.76 | 1.059 | 0.931 | 1.201 |
|  | insulin (N=3126) | 0.798 | 0.949 | 0.99 | 0.919 | 1.067 |
|  | SGLT2 (N=734) | 0.89 | 0.949 | 0.99 | 0.863 | 1.133 |
|  | sulfonylureas (N=5653) | 0.706 | 0.949 | 0.987 | 0.922 | 1.057 |
|  | TZD (N=2295) | 0.28 | 0.746 | 0.954 | 0.877 | 1.038 |
|  | summary (N=NA) | 0.725 | NA | 0.967 | 0.804 | 1.164 |
| IGF2R_rs6917747_A | AGI (N=141) | 0.387 | 0.808 | 1.158 | 0.82 | 1.598 |
|  | biguanides (N=8411) | 0.707 | 0.808 | 1.019 | 0.924 | 1.125 |
|  | DPP4 (N=2509) | 0.688 | 0.808 | 0.981 | 0.891 | 1.078 |
|  | GLP1 (N=902) | 0.474 | 0.808 | 1.057 | 0.907 | 1.226 |
|  | insulin (N=3126) | 0.273 | 0.808 | 1.05 | 0.962 | 1.146 |
|  | SGLT2 (N=734) | 0.676 | 0.808 | 0.966 | 0.819 | 1.133 |
|  | sulfonylureas (N=5653) | 0.851 | 0.851 | 0.992 | 0.916 | 1.075 |
|  | TZD (N=2295) | 0.541 | 0.808 | 1.031 | 0.934 | 1.137 |
|  | summary (N=NA) | 0.891 | NA | 1.014 | 0.829 | 1.241 |
| IGF2R_rs76667637_G | AGI (N=141) | 0.175 | 0.904 | 0.38 | 0.063 | 1.195 |
|  | biguanides (N=8411) | 0.589 | 0.904 | 0.933 | 0.727 | 1.206 |
|  | DPP4 (N=2509) | 0.904 | 0.904 | 1.015 | 0.792 | 1.289 |
|  | GLP1 (N=902) | 0.807 | 0.904 | 1.05 | 0.696 | 1.529 |
|  | insulin (N=3126) | 0.487 | 0.904 | 0.921 | 0.727 | 1.157 |
|  | SGLT2 (N=734) | 0.746 | 0.904 | 1.067 | 0.704 | 1.554 |
|  | sulfonylureas (N=5653) | 0.595 | 0.904 | 0.945 | 0.767 | 1.165 |
|  | TZD (N=2295) | 0.442 | 0.904 | 1.103 | 0.855 | 1.408 |
|  | summary (N=NA) | 0.81 | NA | 0.961 | 0.695 | 1.328 |
| IGF2R_rs77153348_G | AGI (N=141) | 0.087 | 0.347 | 0.611 | 0.331 | 1.03 |
|  | biguanides (N=8411) | 0.731 | 0.836 | 0.978 | 0.863 | 1.11 |
|  | DPP4 (N=2509) | 0.47 | 0.751 | 1.046 | 0.925 | 1.181 |
|  | GLP1 (N=902) | 0.31 | 0.62 | 0.903 | 0.738 | 1.096 |
|  | insulin (N=3126) | 0.08 | 0.347 | 0.901 | 0.801 | 1.011 |
|  | SGLT2 (N=734) | 0.885 | 0.885 | 0.985 | 0.795 | 1.207 |
|  | sulfonylureas (N=5653) | 0.173 | 0.462 | 0.93 | 0.838 | 1.032 |
|  | TZD (N=2295) | 0.652 | 0.836 | 0.971 | 0.853 | 1.103 |
|  | summary (N=NA) | 0.706 | NA | 0.957 | 0.761 | 1.203 |
| IGF2R_rs7750288_G | AGI (N=141) | 0.026 | 0.208 | 1.336 | 1.031 | 1.718 |
|  | biguanides (N=8411) | 0.618 | 0.927 | 1.019 | 0.946 | 1.098 |
|  | DPP4 (N=2509) | 0.427 | 0.927 | 1.03 | 0.958 | 1.106 |
|  | GLP1 (N=902) | 0.654 | 0.927 | 1.027 | 0.914 | 1.152 |
|  | insulin (N=3126) | 0.506 | 0.927 | 0.978 | 0.914 | 1.045 |
|  | SGLT2 (N=734) | 0.696 | 0.927 | 0.976 | 0.862 | 1.102 |
|  | sulfonylureas (N=5653) | 0.962 | 0.962 | 1.001 | 0.942 | 1.065 |
|  | TZD (N=2295) | 0.833 | 0.952 | 0.992 | 0.92 | 1.069 |
|  | summary (N=NA) | 0.926 | NA | 1.008 | 0.846 | 1.202 |
| IGF2R_rs78423775_A | AGI (N=141) | 0.805 | 0.805 | 1.062 | 0.639 | 1.661 |
|  | biguanides (N=8411) | 0.55 | 0.733 | 0.959 | 0.838 | 1.1 |
|  | DPP4 (N=2509) | 0.315 | 0.661 | 1.069 | 0.938 | 1.215 |
|  | GLP1 (N=902) | 0.642 | 0.734 | 0.953 | 0.773 | 1.165 |
|  | insulin (N=3126) | 0.37 | 0.661 | 0.945 | 0.834 | 1.068 |
|  | SGLT2 (N=734) | 0.413 | 0.661 | 1.093 | 0.879 | 1.345 |
|  | sulfonylureas (N=5653) | 0.072 | 0.58 | 1.108 | 0.991 | 1.239 |
|  | TZD (N=2295) | 0.373 | 0.661 | 1.063 | 0.928 | 1.213 |
|  | summary (N=NA) | 0.892 | NA | 1.017 | 0.802 | 1.288 |
| IGF2R_rs78425119_A | AGI (N=141) | 0.963 | 0.963 | 0.988 | 0.567 | 1.599 |
|  | biguanides (N=8411) | 0.157 | 0.471 | 1.109 | 0.962 | 1.283 |
|  | DPP4 (N=2509) | 0.791 | 0.904 | 1.019 | 0.887 | 1.167 |
|  | GLP1 (N=902) | 0.177 | 0.471 | 1.151 | 0.935 | 1.407 |
|  | insulin (N=3126) | 0.147 | 0.471 | 1.097 | 0.967 | 1.242 |
|  | SGLT2 (N=734) | 0.427 | 0.693 | 1.096 | 0.868 | 1.367 |
|  | sulfonylureas (N=5653) | 0.433 | 0.693 | 0.955 | 0.851 | 1.072 |
|  | TZD (N=2295) | 0.571 | 0.762 | 0.959 | 0.83 | 1.105 |
|  | summary (N=NA) | 0.722 | NA | 1.045 | 0.82 | 1.332 |
| IGF2R_rs80254170_G | AGI (N=141) | 0.696 | 0.796 | 0.905 | 0.529 | 1.447 |
|  | biguanides (N=8411) | 0.144 | 0.579 | 1.107 | 0.967 | 1.272 |
|  | DPP4 (N=2509) | 0.964 | 0.964 | 1.003 | 0.879 | 1.141 |
|  | GLP1 (N=902) | 0.27 | 0.721 | 1.117 | 0.914 | 1.356 |
|  | insulin (N=3126) | 0.145 | 0.579 | 1.093 | 0.969 | 1.23 |
|  | SGLT2 (N=734) | 0.693 | 0.796 | 1.045 | 0.836 | 1.291 |
|  | sulfonylureas (N=5653) | 0.516 | 0.796 | 0.964 | 0.864 | 1.076 |
|  | TZD (N=2295) | 0.507 | 0.796 | 0.955 | 0.832 | 1.093 |
|  | summary (N=NA) | 0.741 | NA | 1.041 | 0.821 | 1.319 |
| IGF2R_rs8191725_G | AGI (N=141) | 0.862 | 0.862 | 0.939 | 0.426 | 1.77 |
|  | biguanides (N=8411) | 0.215 | 0.716 | 0.89 | 0.741 | 1.072 |
|  | DPP4 (N=2509) | 0.652 | 0.862 | 0.958 | 0.794 | 1.149 |
|  | GLP1 (N=902) | 0.411 | 0.716 | 0.882 | 0.647 | 1.178 |
|  | insulin (N=3126) | 0.448 | 0.716 | 0.936 | 0.786 | 1.109 |
|  | SGLT2 (N=734) | 0.346 | 0.716 | 1.15 | 0.85 | 1.521 |
|  | sulfonylureas (N=5653) | 0.149 | 0.716 | 0.892 | 0.764 | 1.042 |
|  | TZD (N=2295) | 0.757 | 0.862 | 0.97 | 0.799 | 1.171 |
|  | summary (N=NA) | 0.547 | NA | 0.918 | 0.695 | 1.212 |
| IGF2R_rs8191725_G | AGI (N=141) | 0.299 | 0.478 | 1.186 | 0.85 | 1.619 |
|  | biguanides (N=8411) | 0.006 | 0.046 | 1.146 | 1.041 | 1.263 |
|  | DPP4 (N=2509) | 0.117 | 0.312 | 1.075 | 0.982 | 1.176 |
|  | GLP1 (N=902) | 0.946 | 0.946 | 1.005 | 0.865 | 1.163 |
|  | insulin (N=3126) | 0.766 | 0.875 | 1.013 | 0.93 | 1.102 |
|  | SGLT2 (N=734) | 0.426 | 0.569 | 0.938 | 0.8 | 1.095 |
|  | sulfonylureas (N=5653) | 0.258 | 0.478 | 1.046 | 0.968 | 1.131 |
|  | TZD (N=2295) | 0.052 | 0.208 | 1.097 | 0.999 | 1.205 |
|  | summary (N=NA) | 0.452 | NA | 1.079 | 0.885 | 1.317 |
| IGF2R_rs8191754_G | AGI (N=141) | 0.802 | 0.802 | 1.112 | 0.432 | 2.343 |
|  | biguanides (N=8411) | 0.694 | 0.802 | 0.952 | 0.746 | 1.224 |
|  | DPP4 (N=2509) | 0.801 | 0.802 | 1.031 | 0.81 | 1.3 |
|  | GLP1 (N=902) | 0.617 | 0.802 | 1.098 | 0.75 | 1.561 |
|  | insulin (N=3126) | 0.641 | 0.802 | 1.054 | 0.843 | 1.309 |
|  | SGLT2 (N=734) | 0.347 | 0.802 | 0.81 | 0.507 | 1.227 |
|  | sulfonylureas (N=5653) | 0.73 | 0.802 | 0.965 | 0.788 | 1.182 |
|  | TZD (N=2295) | 0.549 | 0.802 | 1.077 | 0.841 | 1.366 |
|  | summary (N=NA) | 0.945 | NA | 0.989 | 0.718 | 1.361 |
| IGF2R_rs8191871_T | AGI (N=141) | 0.796 | 0.868 | 0.941 | 0.576 | 1.451 |
|  | biguanides (N=8411) | 0.868 | 0.868 | 0.989 | 0.868 | 1.129 |
|  | DPP4 (N=2509) | 0.26 | 0.694 | 0.93 | 0.818 | 1.054 |
|  | GLP1 (N=902) | 0.412 | 0.823 | 1.087 | 0.887 | 1.32 |
|  | insulin (N=3126) | 0.739 | 0.868 | 0.98 | 0.871 | 1.101 |
|  | SGLT2 (N=734) | 0.801 | 0.868 | 0.973 | 0.785 | 1.195 |
|  | sulfonylureas (N=5653) | 0.26 | 0.694 | 1.063 | 0.956 | 1.184 |
|  | TZD (N=2295) | 0.142 | 0.694 | 1.1 | 0.967 | 1.247 |
|  | summary (N=NA) | 0.923 | NA | 1.012 | 0.801 | 1.277 |
| IGF2R_rs879862_T | AGI (N=141) | 0.515 | 0.637 | 1.099 | 0.82 | 1.453 |
|  | biguanides (N=8411) | 0.052 | 0.221 | 0.923 | 0.851 | 1.001 |
|  | DPP4 (N=2509) | 0.407 | 0.637 | 0.967 | 0.894 | 1.046 |
|  | GLP1 (N=902) | 0.278 | 0.556 | 1.071 | 0.945 | 1.211 |
|  | insulin (N=3126) | 0.185 | 0.493 | 0.952 | 0.884 | 1.024 |
|  | SGLT2 (N=734) | 0.055 | 0.221 | 1.134 | 0.996 | 1.288 |
|  | sulfonylureas (N=5653) | 0.558 | 0.637 | 0.98 | 0.917 | 1.048 |
|  | TZD (N=2295) | 0.954 | 0.954 | 0.998 | 0.919 | 1.082 |
|  | summary (N=NA) | 0.697 | NA | 0.964 | 0.803 | 1.158 |
| IGF2R_rs9457795_G | AGI (N=141) | 0.753 | 0.753 | 1.042 | 0.803 | 1.341 |
|  | biguanides (N=8411) | 0.03 | 0.21 | 0.924 | 0.86 | 0.992 |
|  | DPP4 (N=2509) | 0.119 | 0.226 | 0.946 | 0.882 | 1.014 |
|  | GLP1 (N=902) | 0.168 | 0.226 | 1.08 | 0.968 | 1.204 |
|  | insulin (N=3126) | 0.169 | 0.226 | 0.956 | 0.896 | 1.019 |
|  | SGLT2 (N=734) | 0.094 | 0.226 | 1.103 | 0.983 | 1.236 |
|  | sulfonylureas (N=5653) | 0.052 | 0.21 | 0.944 | 0.89 | 1.001 |
|  | TZD (N=2295) | 0.399 | 0.456 | 0.969 | 0.902 | 1.042 |
|  | summary (N=NA) | 0.566 | NA | 0.951 | 0.8 | 1.129 |
| IGF2R_rs998203_T | AGI (N=141) | 0.682 | 0.78 | 1.053 | 0.821 | 1.344 |
|  | biguanides (N=8411) | 0.372 | 0.732 | 0.969 | 0.905 | 1.038 |
|  | DPP4 (N=2509) | 0.854 | 0.854 | 1.006 | 0.942 | 1.075 |
|  | GLP1 (N=902) | 0.67 | 0.78 | 0.977 | 0.879 | 1.086 |
|  | insulin (N=3126) | 0.254 | 0.732 | 1.036 | 0.975 | 1.101 |
|  | SGLT2 (N=734) | 0.458 | 0.732 | 1.043 | 0.933 | 1.164 |
|  | sulfonylureas (N=5653) | 0.412 | 0.732 | 1.024 | 0.968 | 1.083 |
|  | TZD (N=2295) | 0.032 | 0.255 | 1.078 | 1.006 | 1.154 |
|  | summary (N=NA) | 0.927 | NA | 1.008 | 0.852 | 1.192 |
| MAS1_rs12207188_T | AGI (N=141) | 0.841 | 0.962 | 0.945 | 0.521 | 1.579 |
|  | biguanides (N=8411) | 0.669 | 0.962 | 1.032 | 0.894 | 1.195 |
|  | DPP4 (N=2509) | 0.838 | 0.962 | 1.015 | 0.88 | 1.167 |
|  | GLP1 (N=902) | 0.984 | 0.984 | 0.998 | 0.797 | 1.238 |
|  | insulin (N=3126) | 0.277 | 0.962 | 1.074 | 0.943 | 1.221 |
|  | SGLT2 (N=734) | 0.3 | 0.962 | 1.13 | 0.891 | 1.417 |
|  | sulfonylureas (N=5653) | 0.505 | 0.962 | 1.041 | 0.925 | 1.173 |
|  | TZD (N=2295) | 0.565 | 0.962 | 1.044 | 0.901 | 1.205 |
|  | summary (N=NA) | 0.753 | NA | 1.04 | 0.814 | 1.329 |
| MAS1_rs170219_G | AGI (N=141) | 0.693 | 0.693 | 1.053 | 0.813 | 1.352 |
|  | biguanides (N=8411) | 0.026 | 0.102 | 0.922 | 0.859 | 0.99 |
|  | DPP4 (N=2509) | 0.133 | 0.213 | 0.948 | 0.885 | 1.016 |
|  | GLP1 (N=902) | 0.34 | 0.389 | 1.055 | 0.945 | 1.176 |
|  | insulin (N=3126) | 0.119 | 0.213 | 0.95 | 0.891 | 1.013 |
|  | SGLT2 (N=734) | 0.263 | 0.351 | 1.068 | 0.951 | 1.197 |
|  | sulfonylureas (N=5653) | 0.021 | 0.102 | 0.933 | 0.88 | 0.99 |
|  | TZD (N=2295) | 0.132 | 0.213 | 0.946 | 0.88 | 1.017 |
|  | summary (N=NA) | 0.505 | NA | 0.943 | 0.794 | 1.12 |
| MAS1_rs220721_T | AGI (N=141) | 0.344 | 0.458 | 0.863 | 0.631 | 1.16 |
|  | biguanides (N=8411) | 0.07 | 0.204 | 0.929 | 0.858 | 1.006 |
|  | DPP4 (N=2509) | 0.428 | 0.49 | 0.969 | 0.896 | 1.047 |
|  | GLP1 (N=902) | 0.948 | 0.948 | 1.004 | 0.887 | 1.135 |
|  | insulin (N=3126) | 0.195 | 0.312 | 0.953 | 0.886 | 1.025 |
|  | SGLT2 (N=734) | 0.166 | 0.312 | 1.095 | 0.962 | 1.243 |
|  | sulfonylureas (N=5653) | 0.006 | 0.045 | 0.911 | 0.853 | 0.973 |
|  | TZD (N=2295) | 0.076 | 0.204 | 0.929 | 0.855 | 1.008 |
|  | summary (N=NA) | 0.498 | NA | 0.939 | 0.783 | 1.126 |
| MAS1_rs220722_A | AGI (N=141) | 0.578 | 0.827 | 0.86 | 0.484 | 1.413 |
|  | biguanides (N=8411) | 0.614 | 0.827 | 1.037 | 0.902 | 1.196 |
|  | DPP4 (N=2509) | 0.827 | 0.827 | 0.985 | 0.858 | 1.128 |
|  | GLP1 (N=902) | 0.281 | 0.827 | 0.887 | 0.709 | 1.098 |
|  | insulin (N=3126) | 0.555 | 0.827 | 0.962 | 0.847 | 1.092 |
|  | SGLT2 (N=734) | 0.478 | 0.827 | 0.918 | 0.72 | 1.155 |
|  | sulfonylureas (N=5653) | 0.777 | 0.827 | 1.017 | 0.907 | 1.141 |
|  | TZD (N=2295) | 0.725 | 0.827 | 1.025 | 0.89 | 1.177 |
|  | summary (N=NA) | 0.971 | NA | 1.004 | 0.789 | 1.279 |
| MAS1_rs220722_A | AGI (N=141) | 0.869 | 0.945 | 1.086 | 0.337 | 2.522 |
|  | biguanides (N=8411) | 0.789 | 0.945 | 0.961 | 0.724 | 1.293 |
|  | DPP4 (N=2509) | 0.249 | 0.945 | 1.172 | 0.889 | 1.526 |
|  | GLP1 (N=902) | 0.161 | 0.945 | 0.698 | 0.406 | 1.12 |
|  | insulin (N=3126) | 0.58 | 0.945 | 0.928 | 0.708 | 1.203 |
|  | SGLT2 (N=734) | 0.945 | 0.945 | 1.016 | 0.615 | 1.578 |
|  | sulfonylureas (N=5653) | 0.771 | 0.945 | 0.965 | 0.762 | 1.224 |
|  | TZD (N=2295) | 0.848 | 0.945 | 0.972 | 0.72 | 1.291 |
|  | summary (N=NA) | 0.861 | NA | 0.97 | 0.686 | 1.371 |
| MAS1_rs220733_A | AGI (N=141) | 0.297 | 0.911 | 1.51 | 0.632 | 3.043 |
|  | biguanides (N=8411) | 0.8 | 0.995 | 0.967 | 0.751 | 1.257 |
|  | DPP4 (N=2509) | 0.466 | 0.932 | 1.097 | 0.851 | 1.401 |
|  | GLP1 (N=902) | 0.271 | 0.911 | 0.783 | 0.494 | 1.185 |
|  | insulin (N=3126) | 0.943 | 0.995 | 1.009 | 0.795 | 1.271 |
|  | SGLT2 (N=734) | 0.798 | 0.995 | 1.057 | 0.674 | 1.58 |
|  | sulfonylureas (N=5653) | 0.342 | 0.911 | 1.109 | 0.896 | 1.375 |
|  | TZD (N=2295) | 0.995 | 0.995 | 1.001 | 0.765 | 1.294 |
|  | summary (N=NA) | 0.911 | NA | 1.019 | 0.735 | 1.413 |
| MAS1_rs910173_G | AGI (N=141) | 0.033 | 0.267 | 1.46 | 1.015 | 2.041 |
|  | biguanides (N=8411) | 0.289 | 0.727 | 0.942 | 0.844 | 1.053 |
|  | DPP4 (N=2509) | 0.364 | 0.727 | 0.951 | 0.853 | 1.059 |
|  | GLP1 (N=902) | 0.123 | 0.492 | 1.139 | 0.963 | 1.34 |
|  | insulin (N=3126) | 0.749 | 0.916 | 0.984 | 0.891 | 1.086 |
|  | SGLT2 (N=734) | 0.91 | 0.916 | 1.01 | 0.842 | 1.203 |
|  | sulfonylureas (N=5653) | 0.827 | 0.916 | 1.01 | 0.923 | 1.106 |
|  | TZD (N=2295) | 0.916 | 0.916 | 1.006 | 0.899 | 1.123 |
|  | summary (N=NA) | 0.868 | NA | 0.982 | 0.793 | 1.216 |

References

1. Action to Control Cardiovascular Risk in Diabetes Study Group *et al.* Effects of intensive glucose lowering in type 2 diabetes. *N. Engl. J. Med.* **358**, 2545–2559 (2008).
